# Supplementary material for: Synthesis, Biological Evaluation, and In Silico Studies of New Acetylcholinesterase Inhibitors Based on Quinoxaline Scaffold
Source: Molecules. 2021 Aug 12;26(16):4895. doi: 10.3390/molecules26164895 (PMC8400540; doi:10.3390/molecules26164895)
Supplement: Supplementary file 1 [file molecules-26-04895-s001.zip › Supplementary-revised/supplementary Materials 07-08-21.pdf]

Supporting Information

Synthesis, Biological Evaluation, and In Silico Studies of New Acetylcholinesterase Inhibitors Based on Quinoxaline Scaffold

Paptawan Suwanhom, Jirakrit Saetang, Pasarat Khongkow, Teerapat Nualnoi, Varomyalin Tipmanee and Luelak Lomlim

- Nuclear magnetic resonance spectra for the synthesized quinoxaline and its derivatives

- IC_{50} curves for the inhibition of *Hu*AChE and *eq*BChE by quinoxaline derivatives (3a-6c), tacrine and galanthamine

- 2D Figure indicated interaction between synthesized compound and the amino acids at AChE binding site

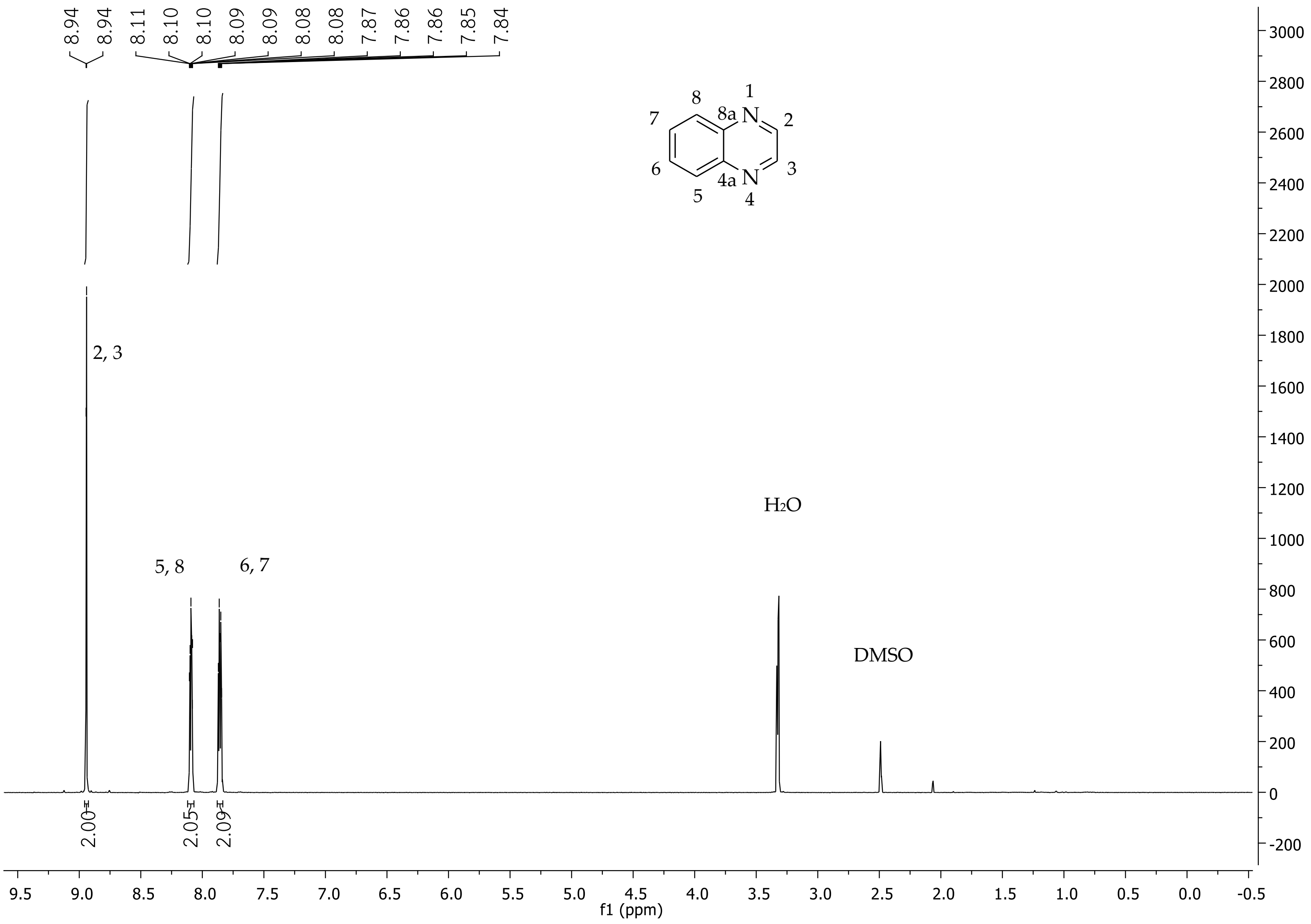

Figure 1. ^1H-NMR spectra of Quinoxaline (**3a**).

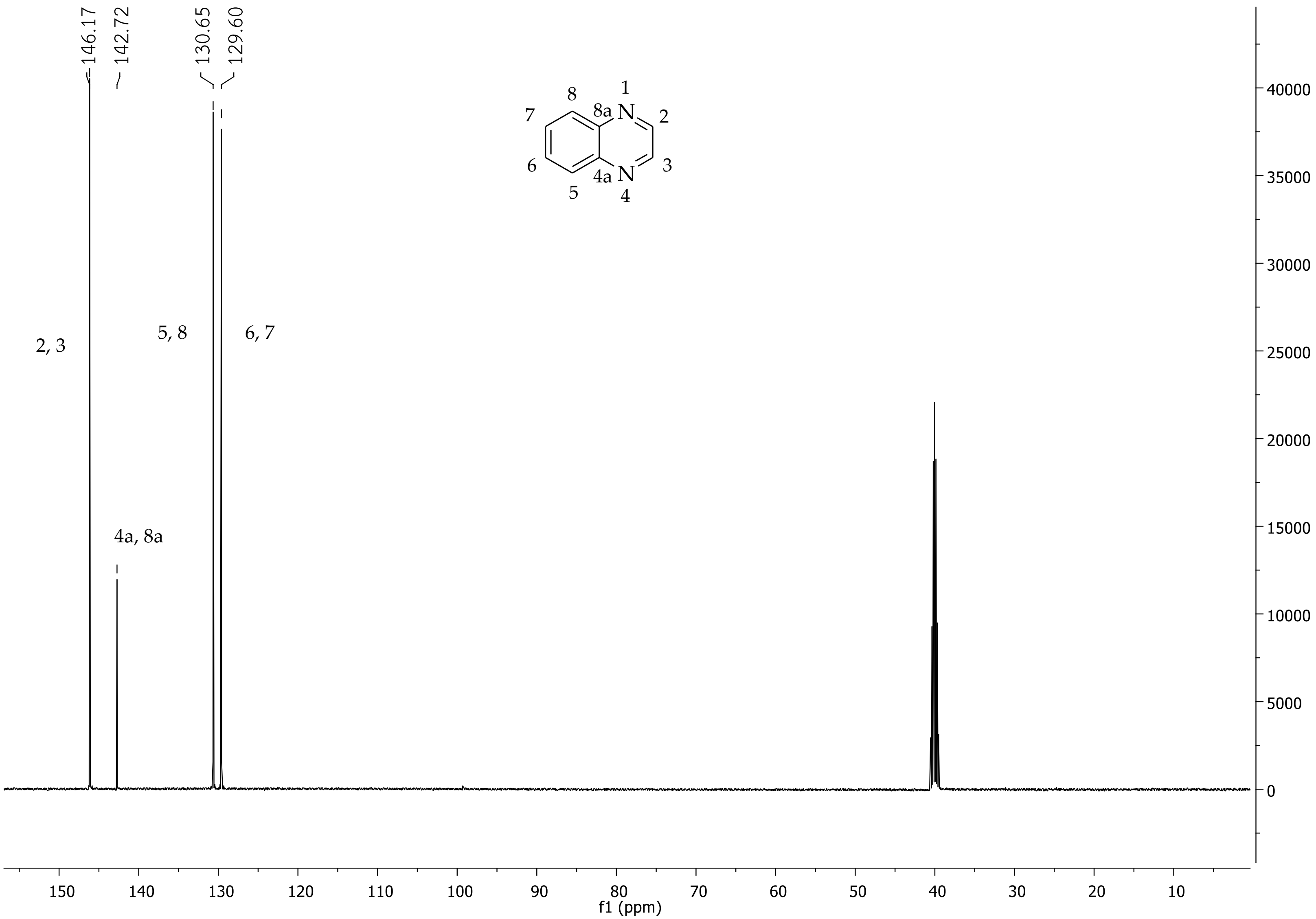

Figure 2. ^{13}C-NMR spectra of Quinoxaline (**3a**).

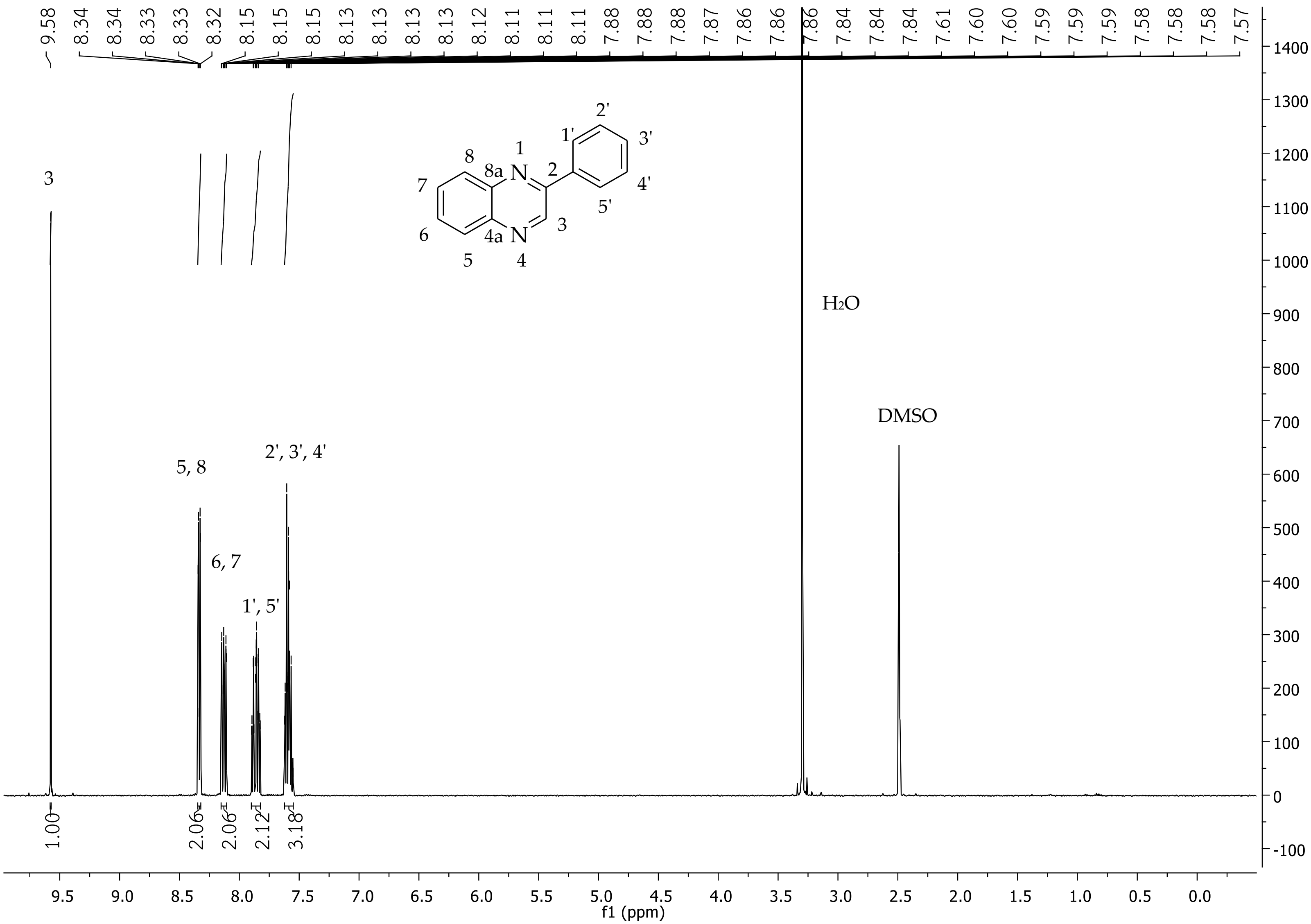

Figure 3. ^{1}H-NMR spectra of 2-Phenylquinoxaline (**3b**).

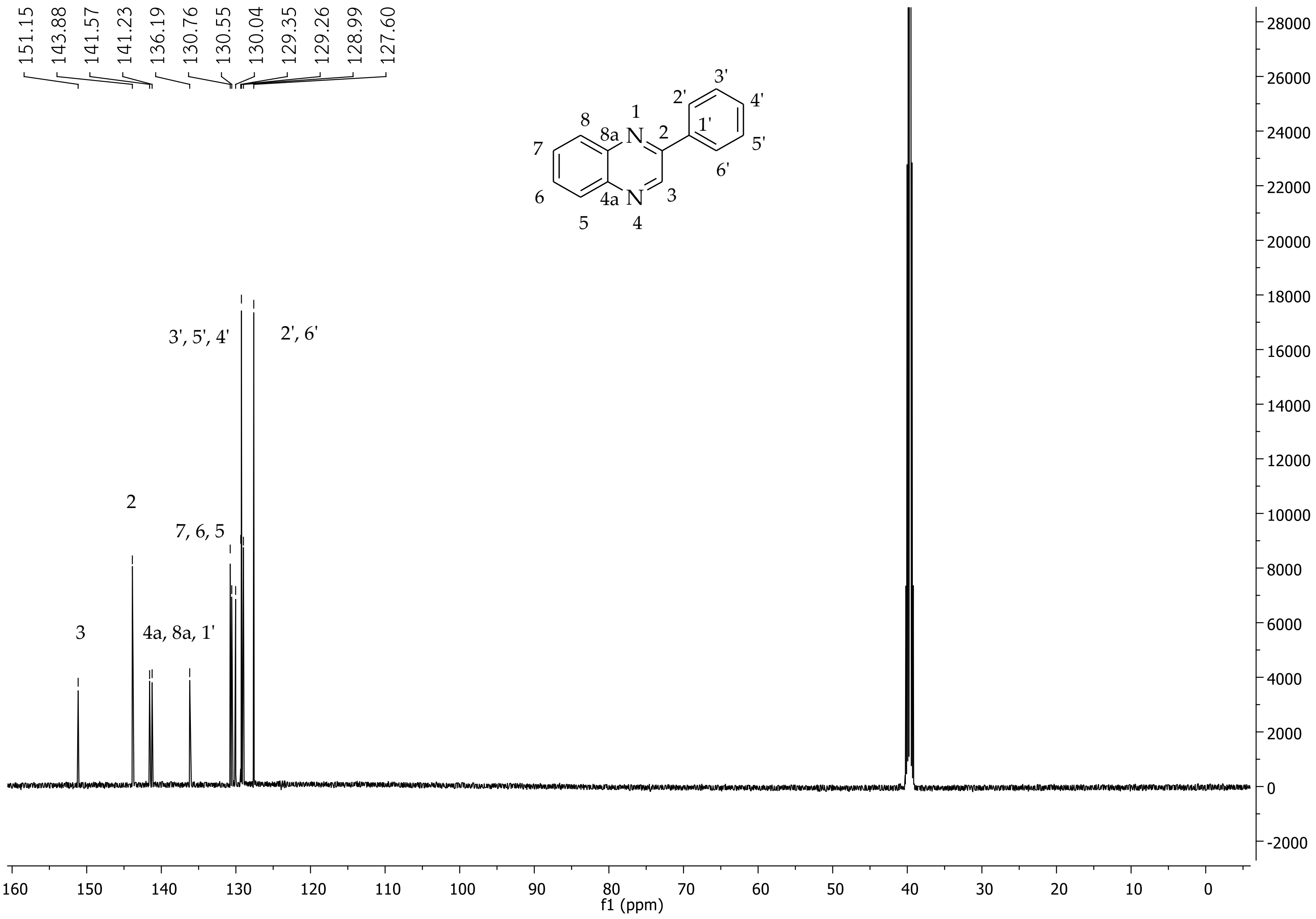

Figure 4. ^{13}C-NMR spectra of 2-Phenylquinoxaline (**3b**).

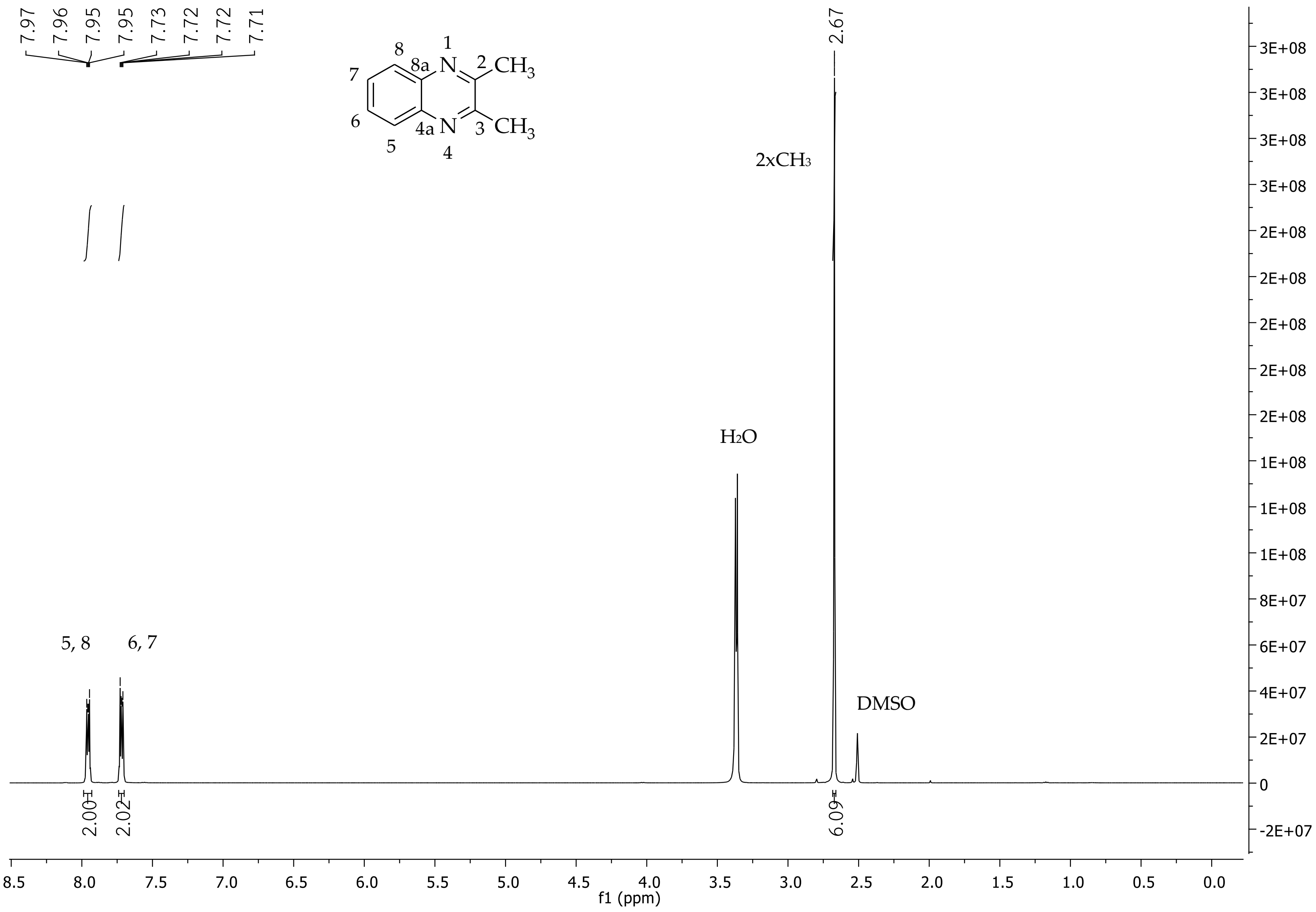

Figure 5. ^{1}H-NMR spectra of 2,3-Dimethylquinoxaline (**3c**).

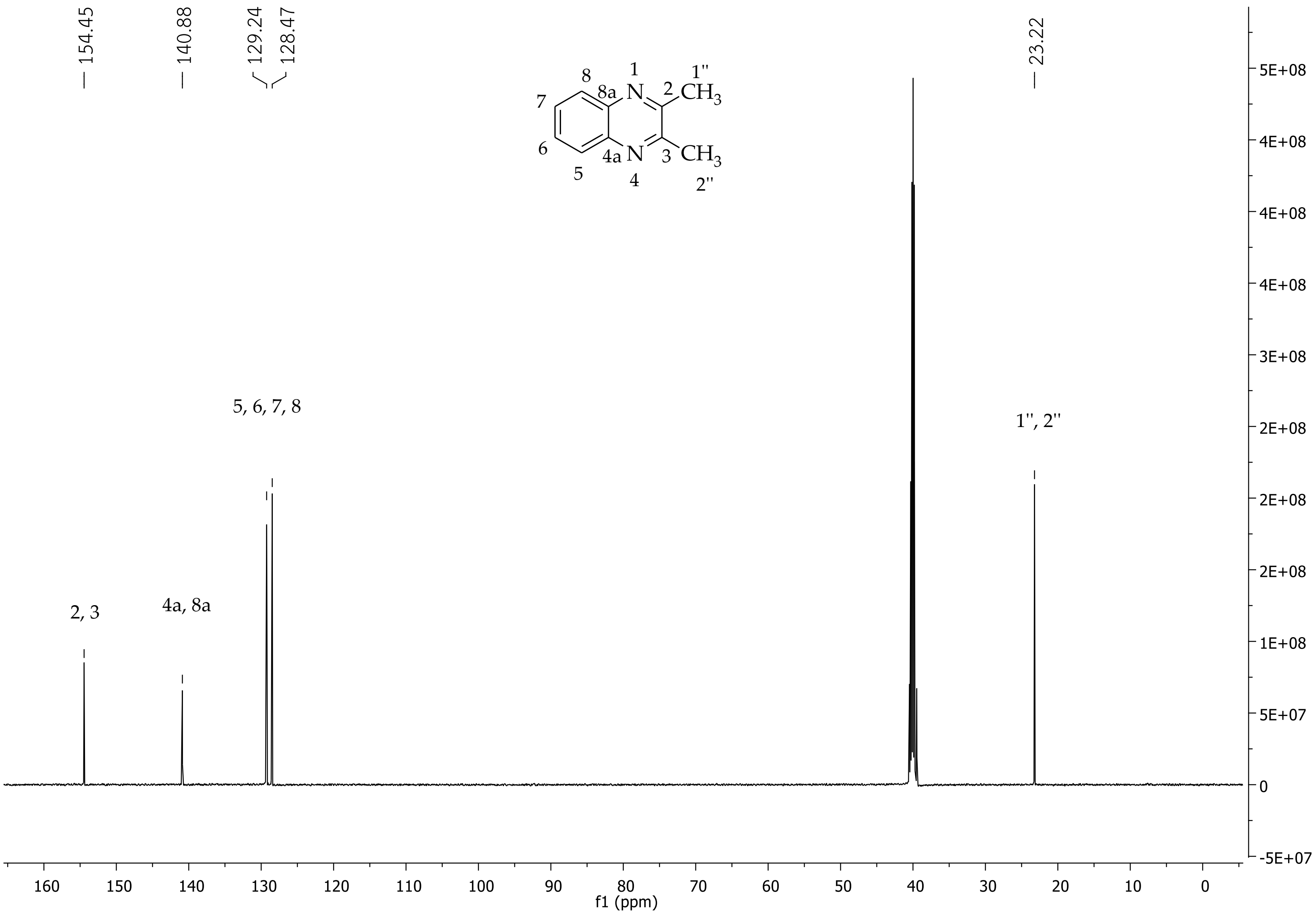

Figure 6. ^{13}C-NMR spectra of 2,3-Dimethylquinoxaline (**3c**).

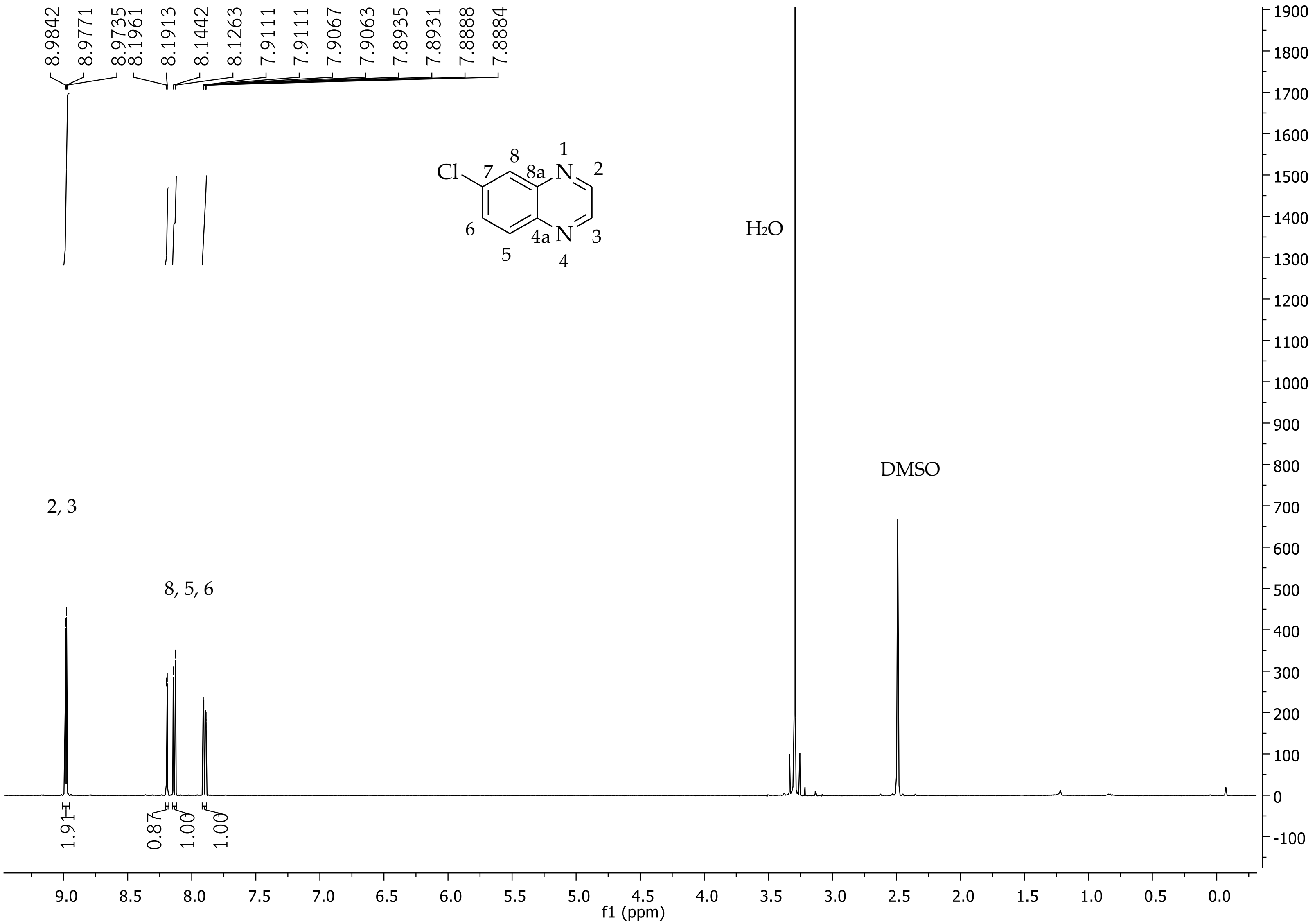

Figure 7. ^{1}H-NMR spectra of 6-Chloroquinoxaline (**4a**).

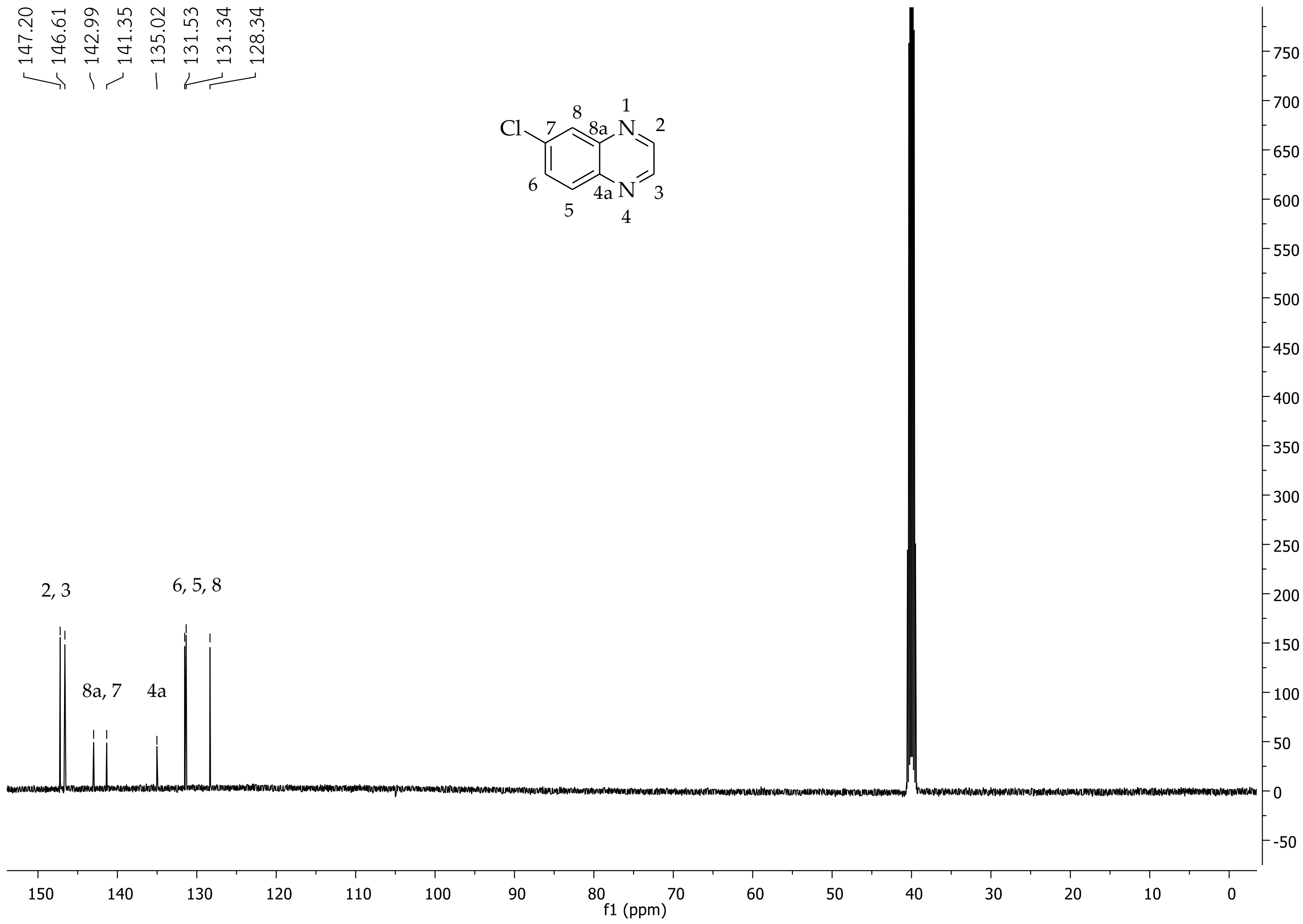

Figure 8. ^{13}C-NMR spectra of 6-Chloroquinoxaline (**4a**).

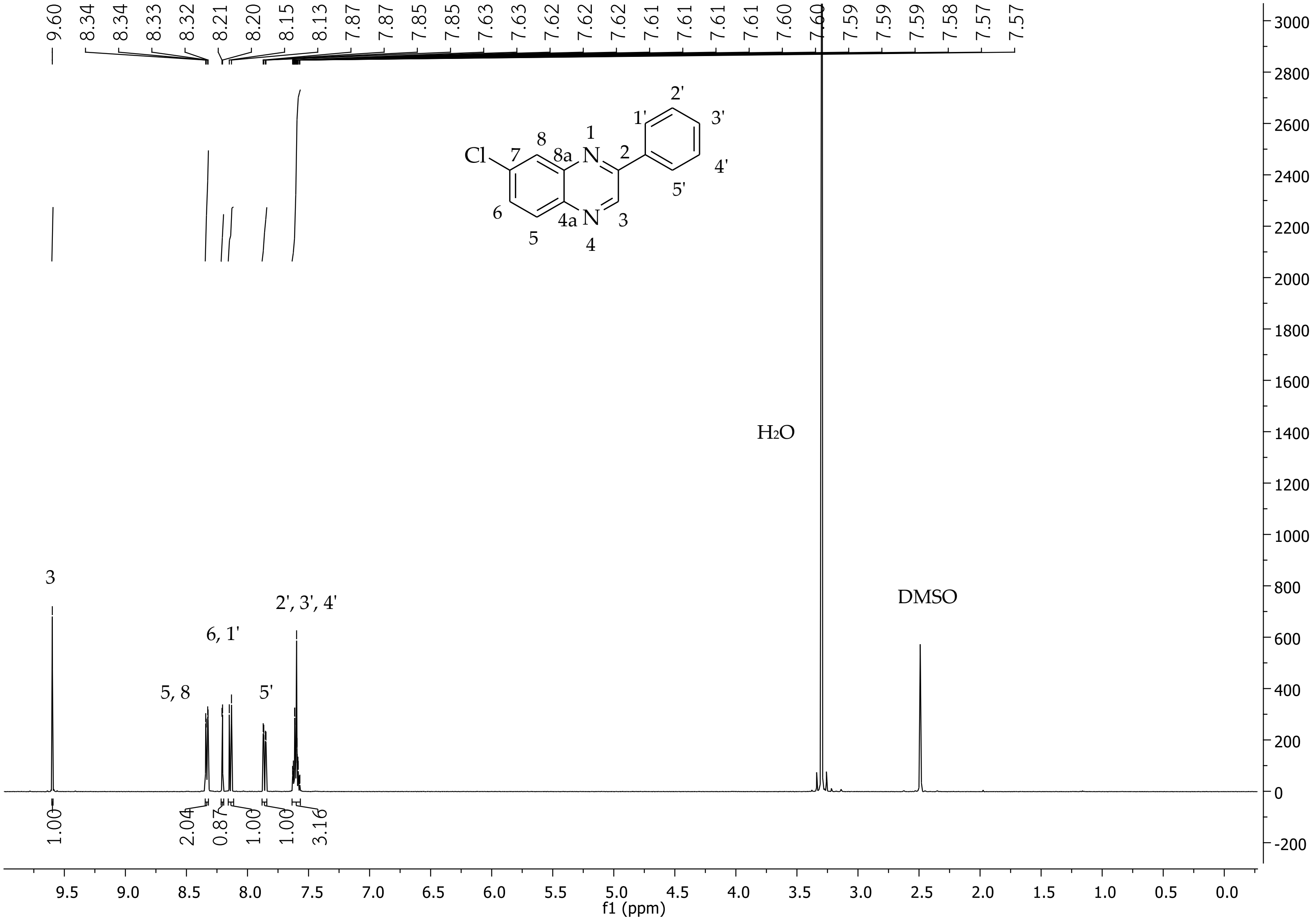

Figure 9. ^{1}H-NMR spectra of 7-Chloro-2-phenylquinoxaline (**4b**).

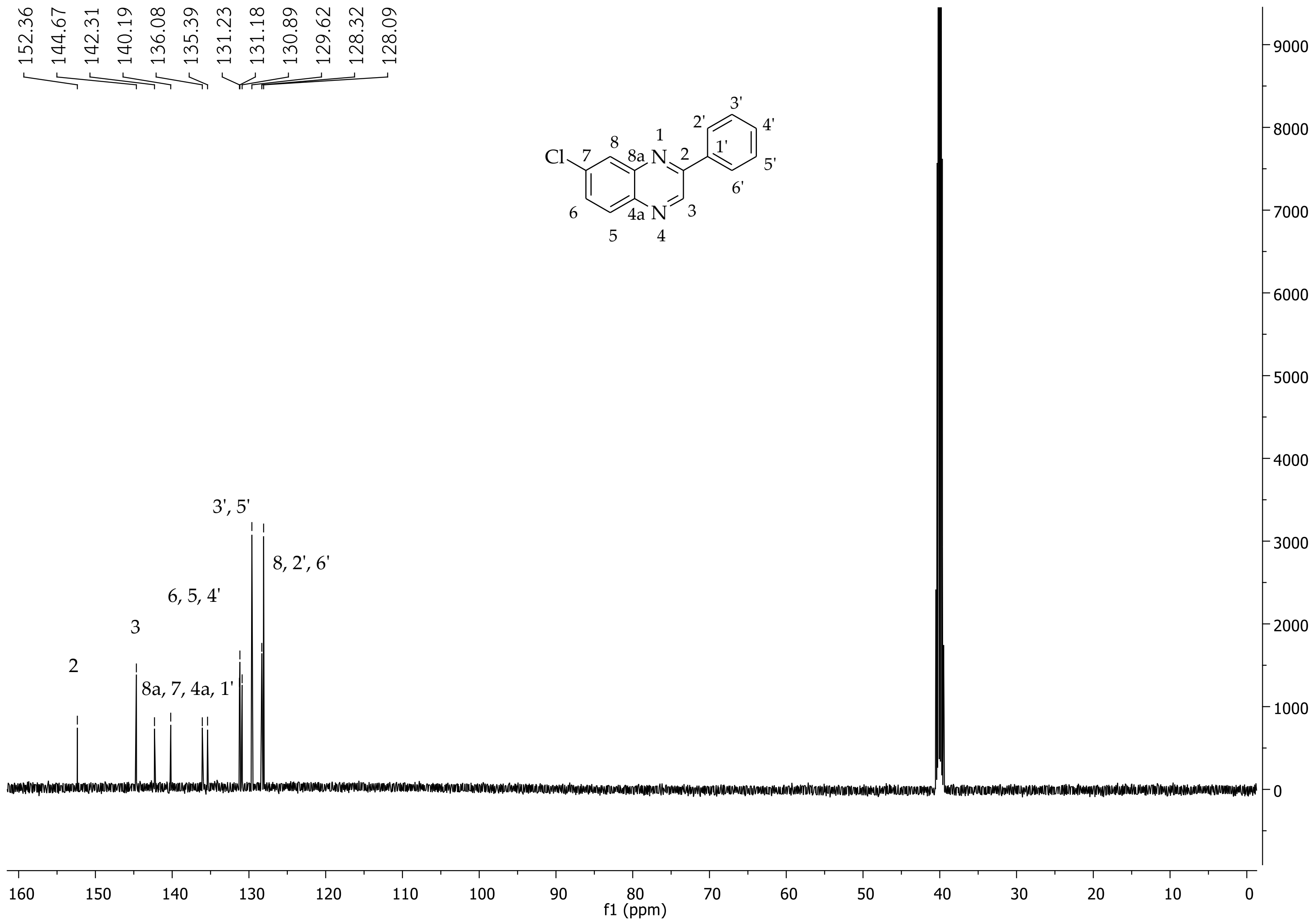

Figure 10. ^{13}C-NMR spectra of 7-Chloro-2-phenylquinoxaline (**4b**).

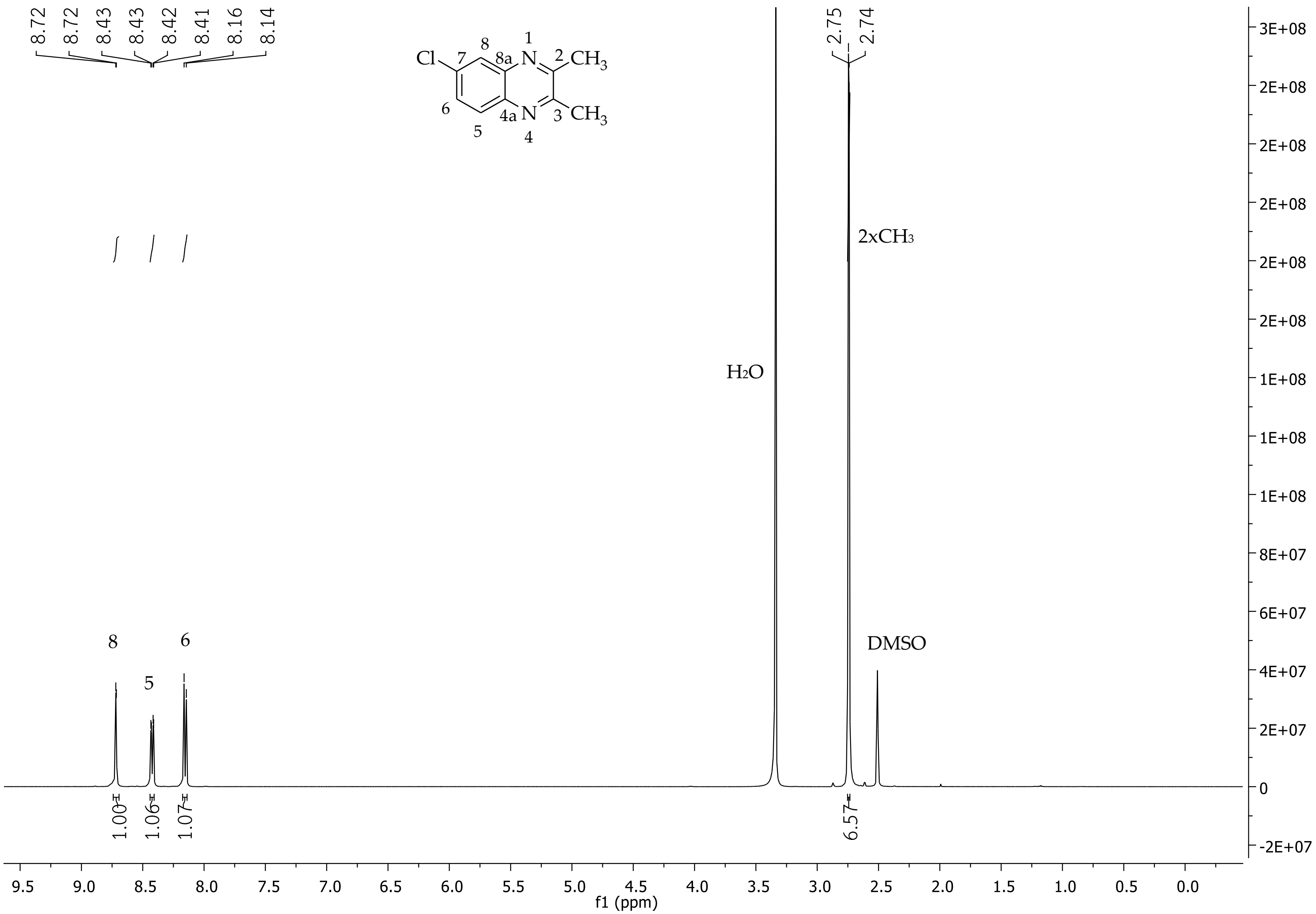

Figure 11. ^{1}H-NMR spectra of 6-Chloro-2,3-dimethylquinoxaline (**4c**).

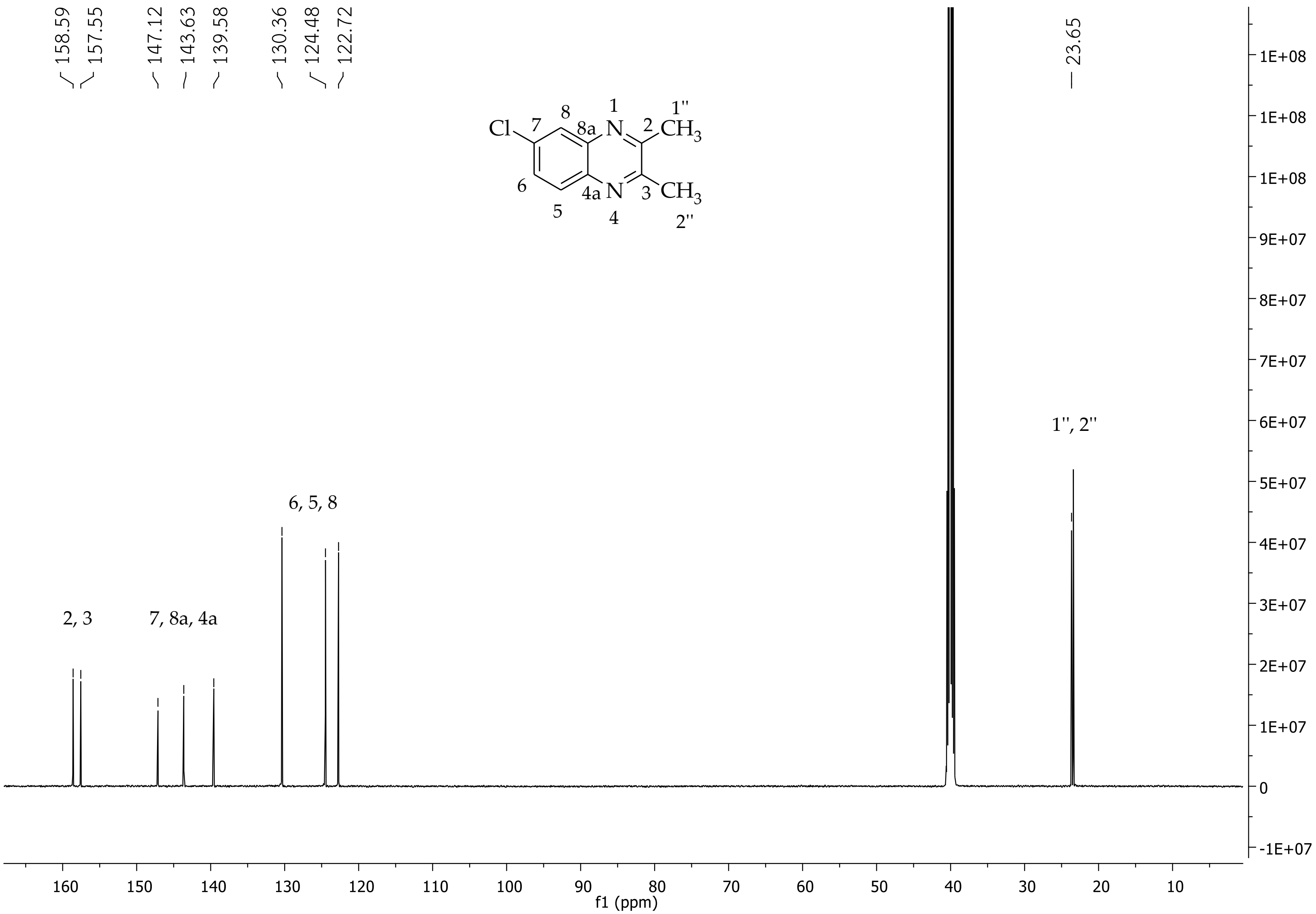

Figure 12. ^{13}C-NMR spectra of 6-Chloro-2,3-dimethylquinoxaline (**4c**).

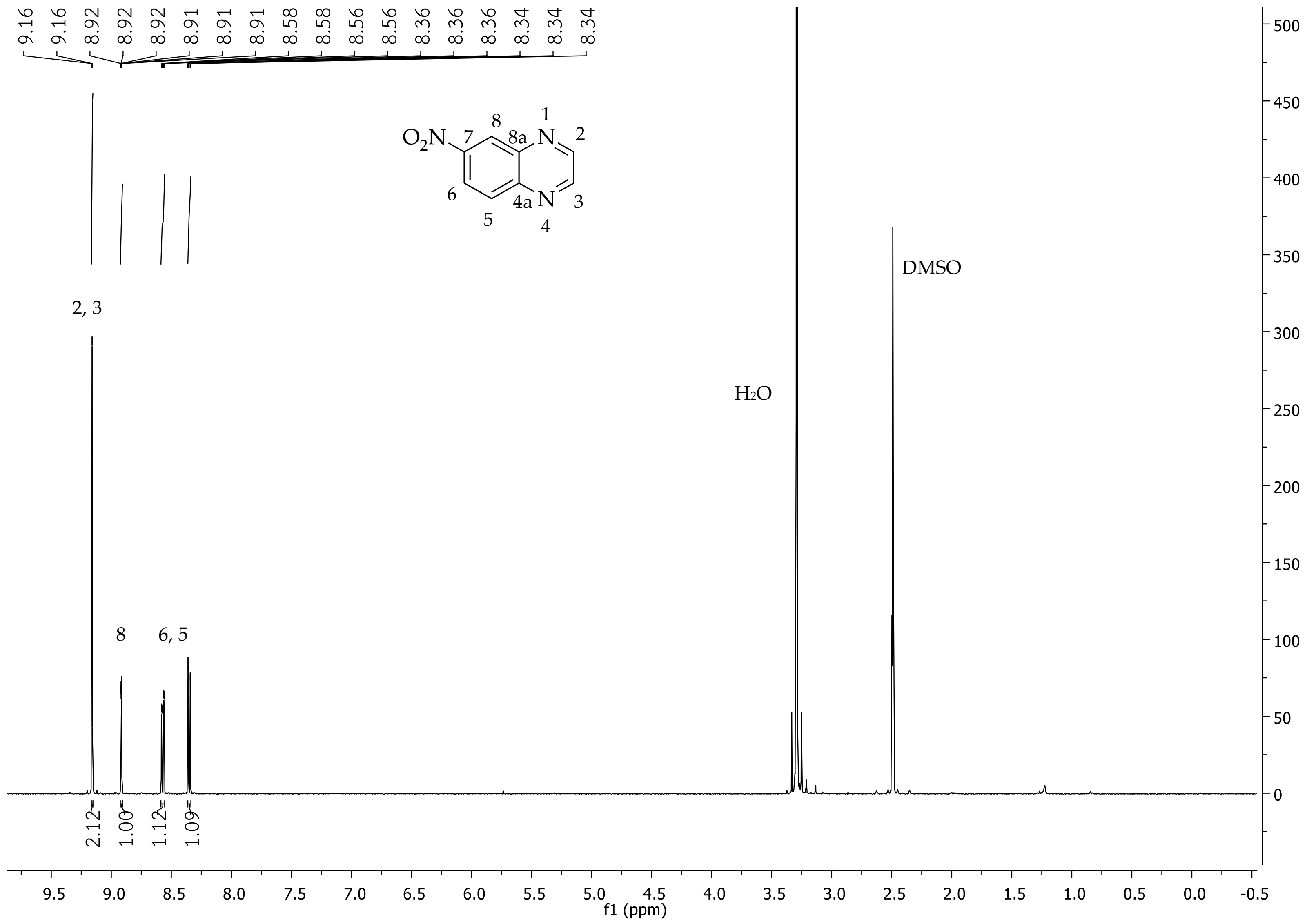

Figure 13. ^{1}H-NMR spectra of 6-Nitroquinoxaline (**5a**).

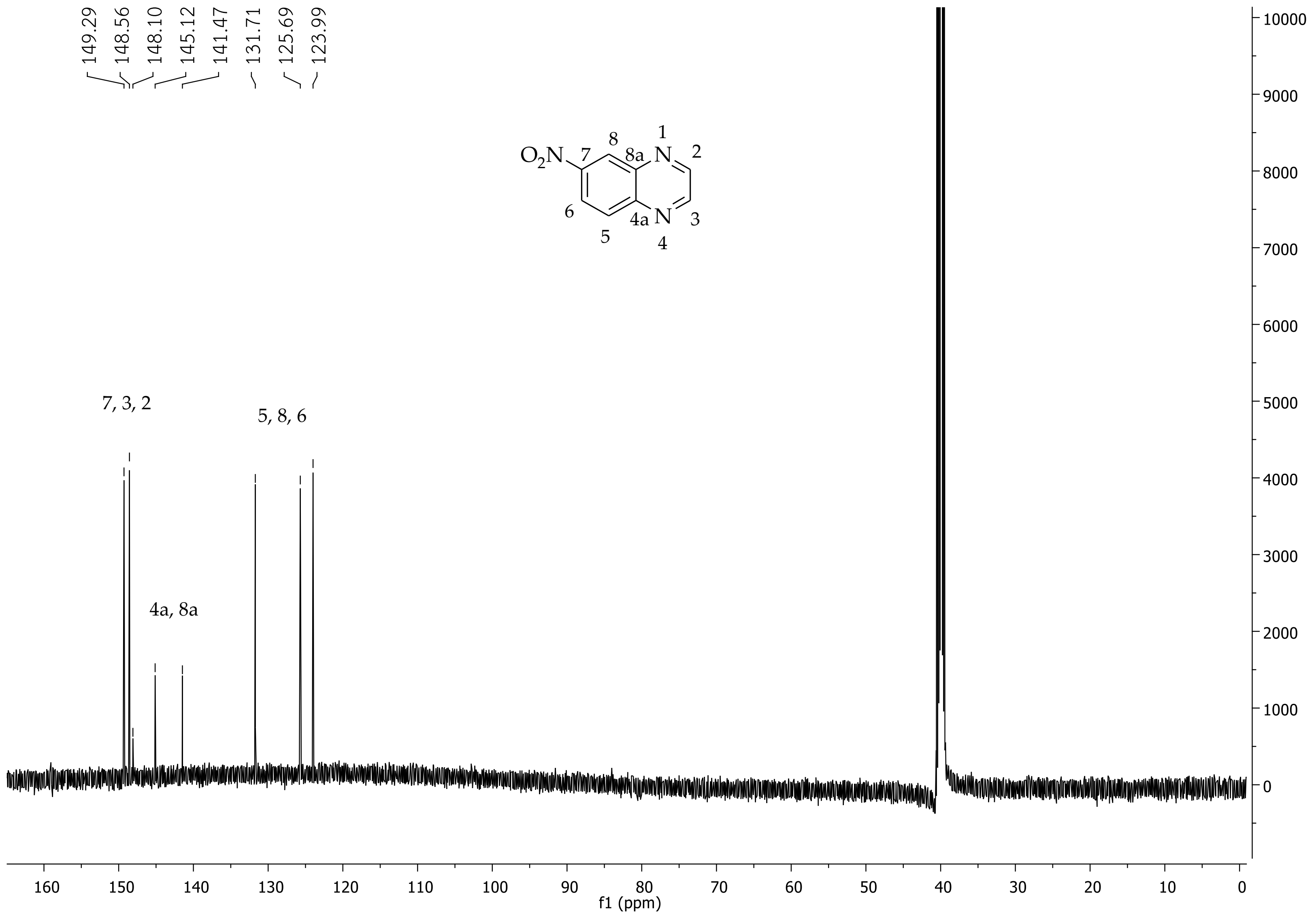

Figure 14. ^{13}C-NMR spectra of 6-Nitroquinoxaline (**5a**).

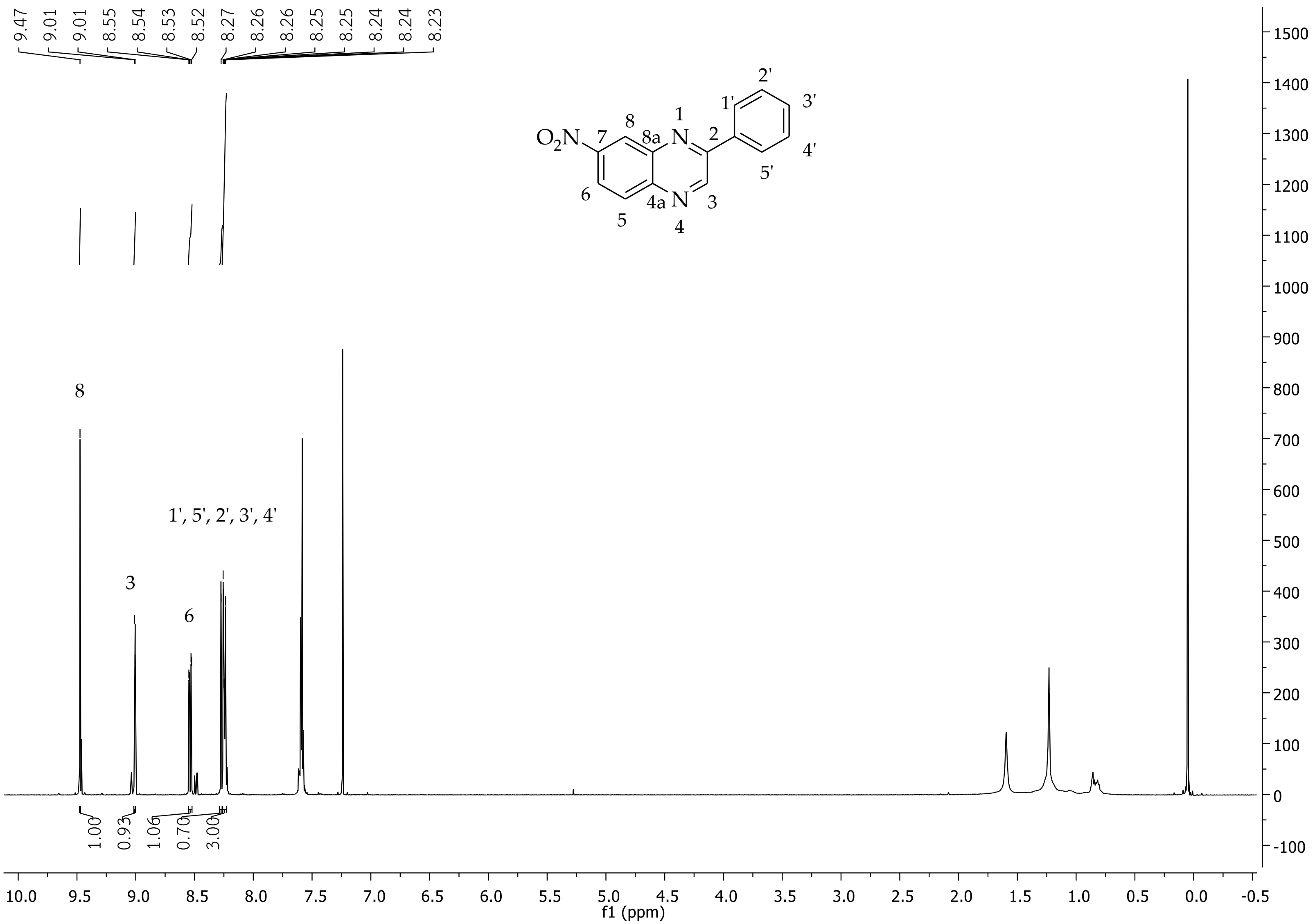

Figure 15. ^{1}H-NMR spectra of 7-Nitro-2-phenylquinoxaline (**5b**).

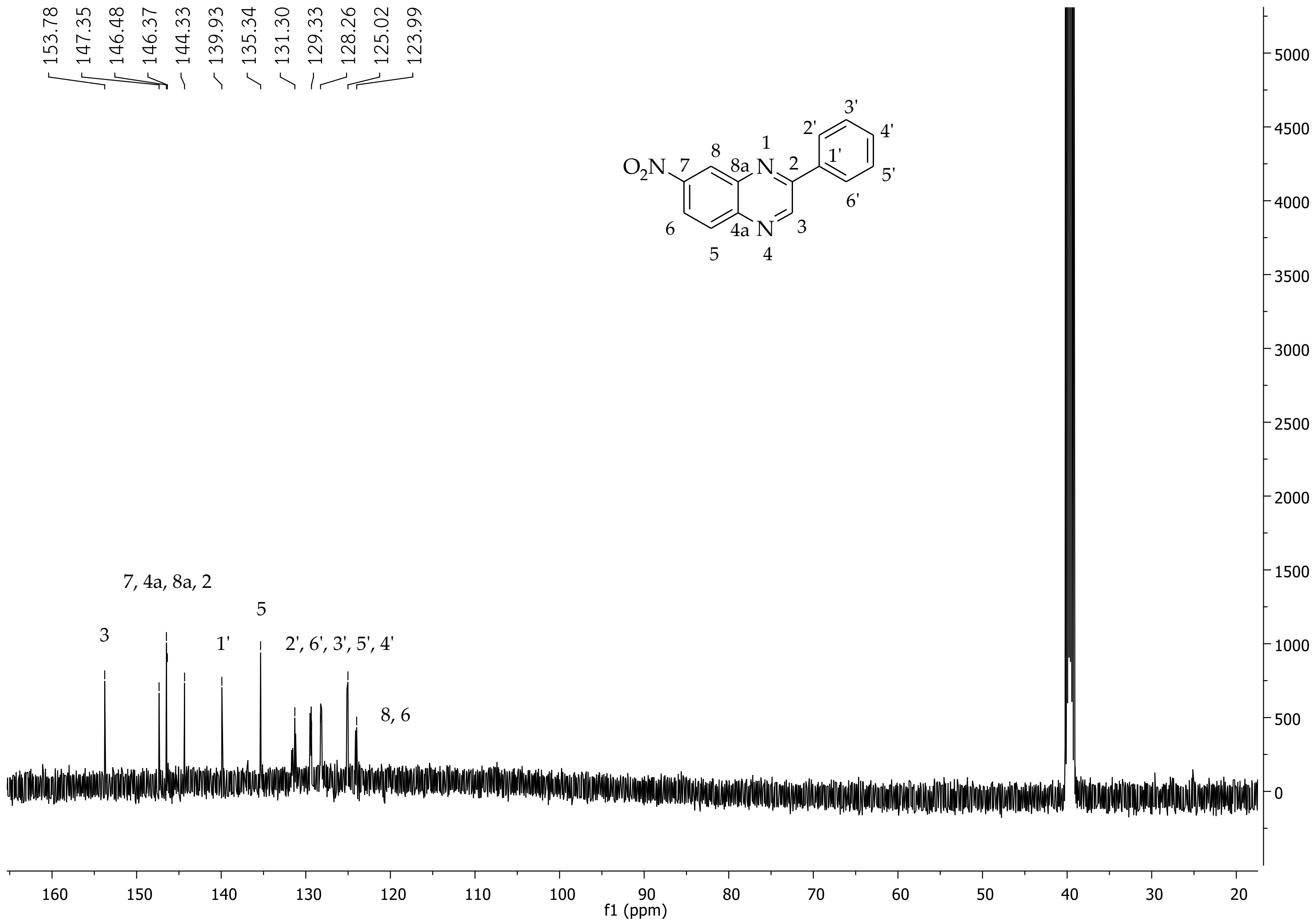

Figure 16. ^{13}C-NMR spectra of 7-Nitro-2-phenylquinoxaline (**5b**).

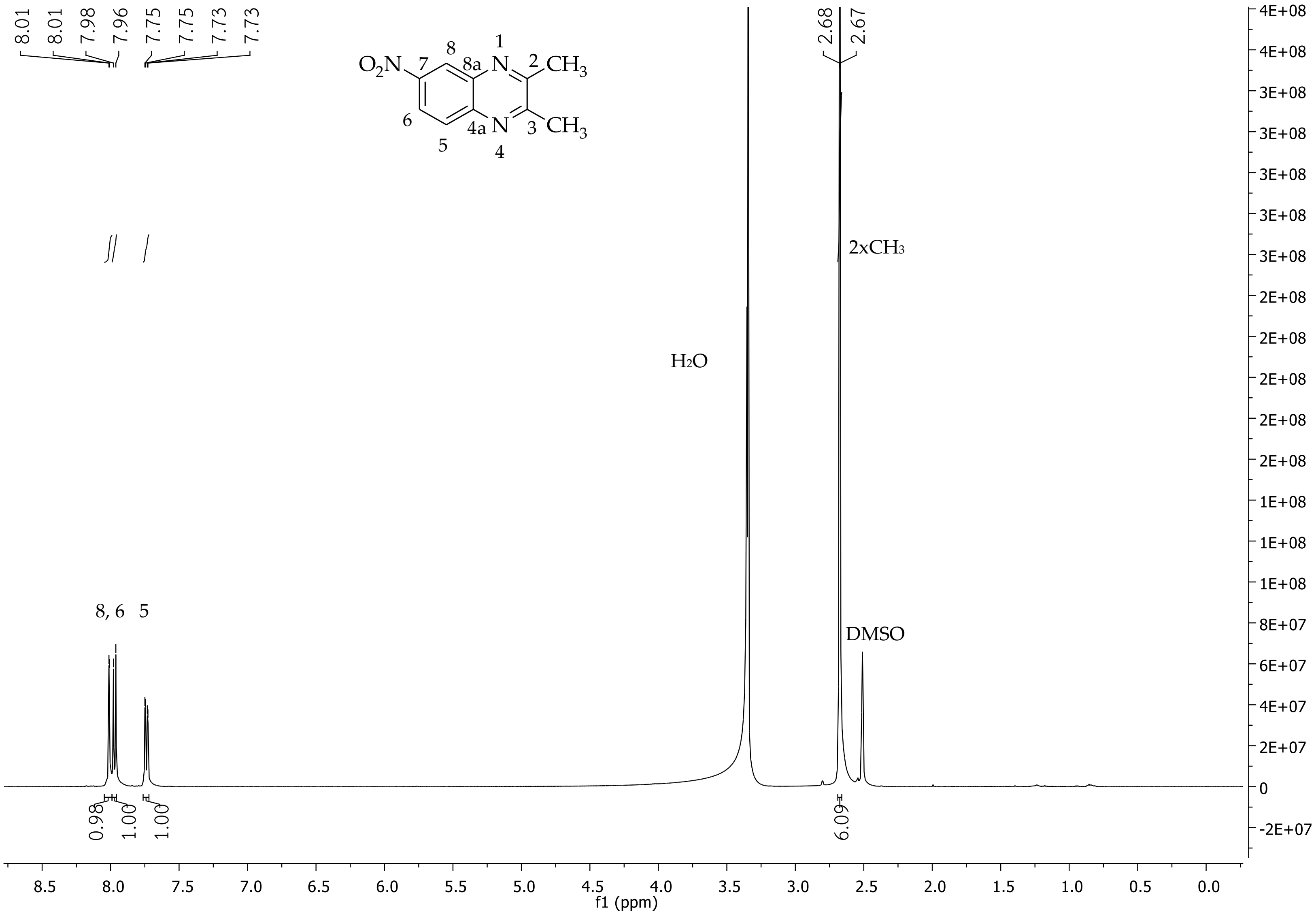

Figure 17. ^{1}H-NMR spectra of 2,3-Dimethyl-6-nitroquinoxaline (**5c**).

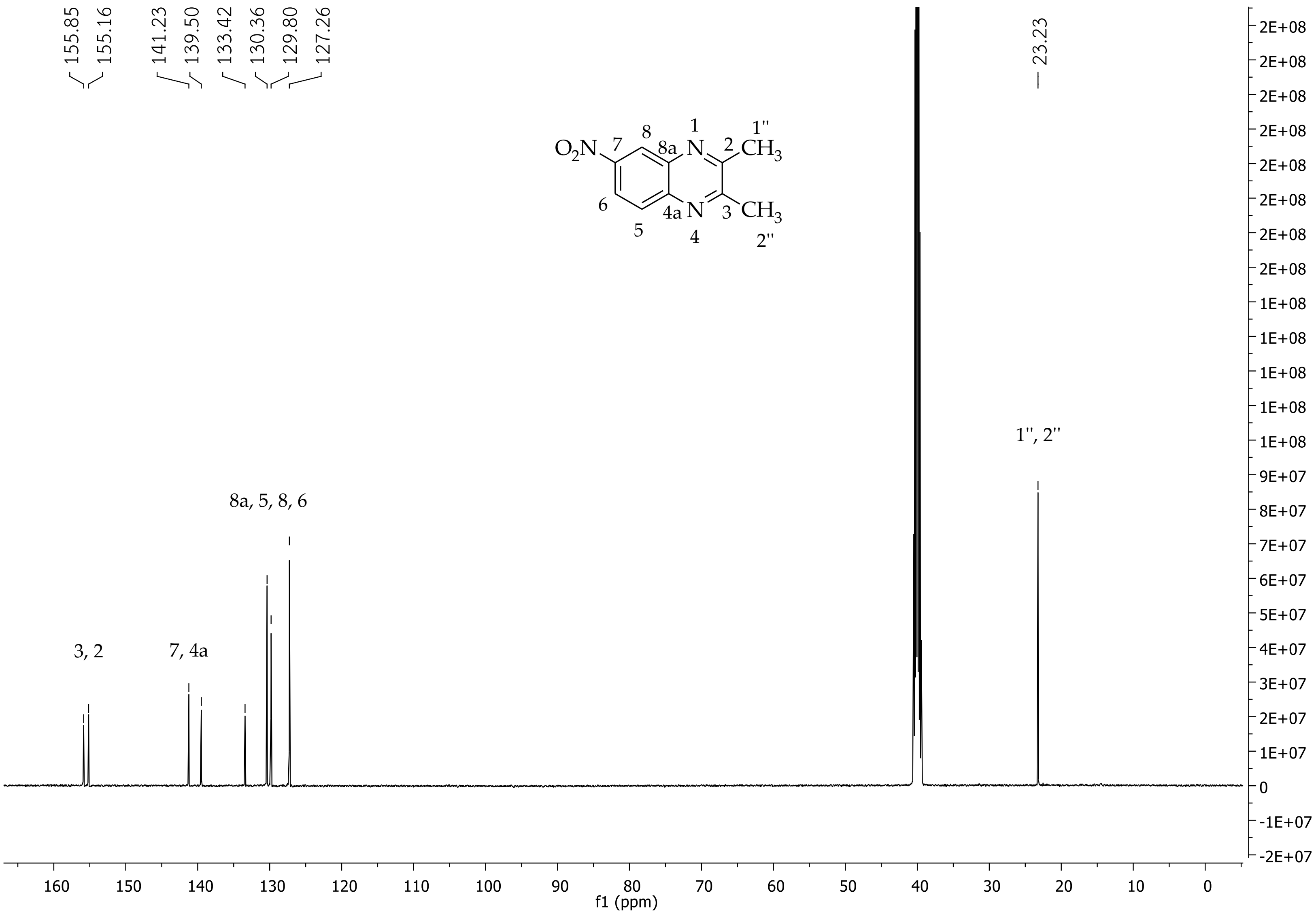

Figure 18. ^{13}C-NMR spectra of 2,3-Dimethyl-6-nitroquinoxaline (**5c**).

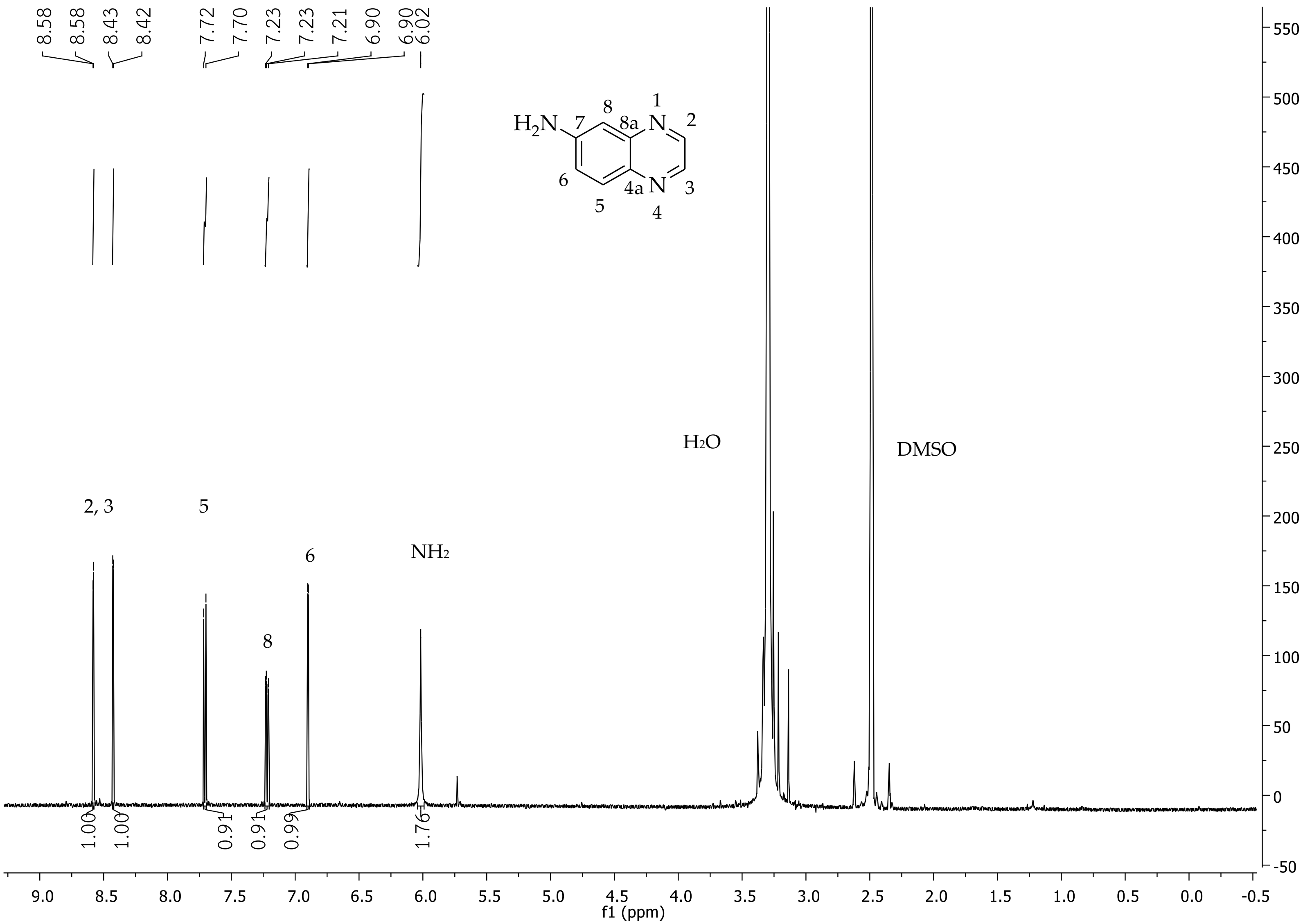

Figure 19. ^{1}H-NMR spectra of Quinoxalin-6-amine (**6a**).

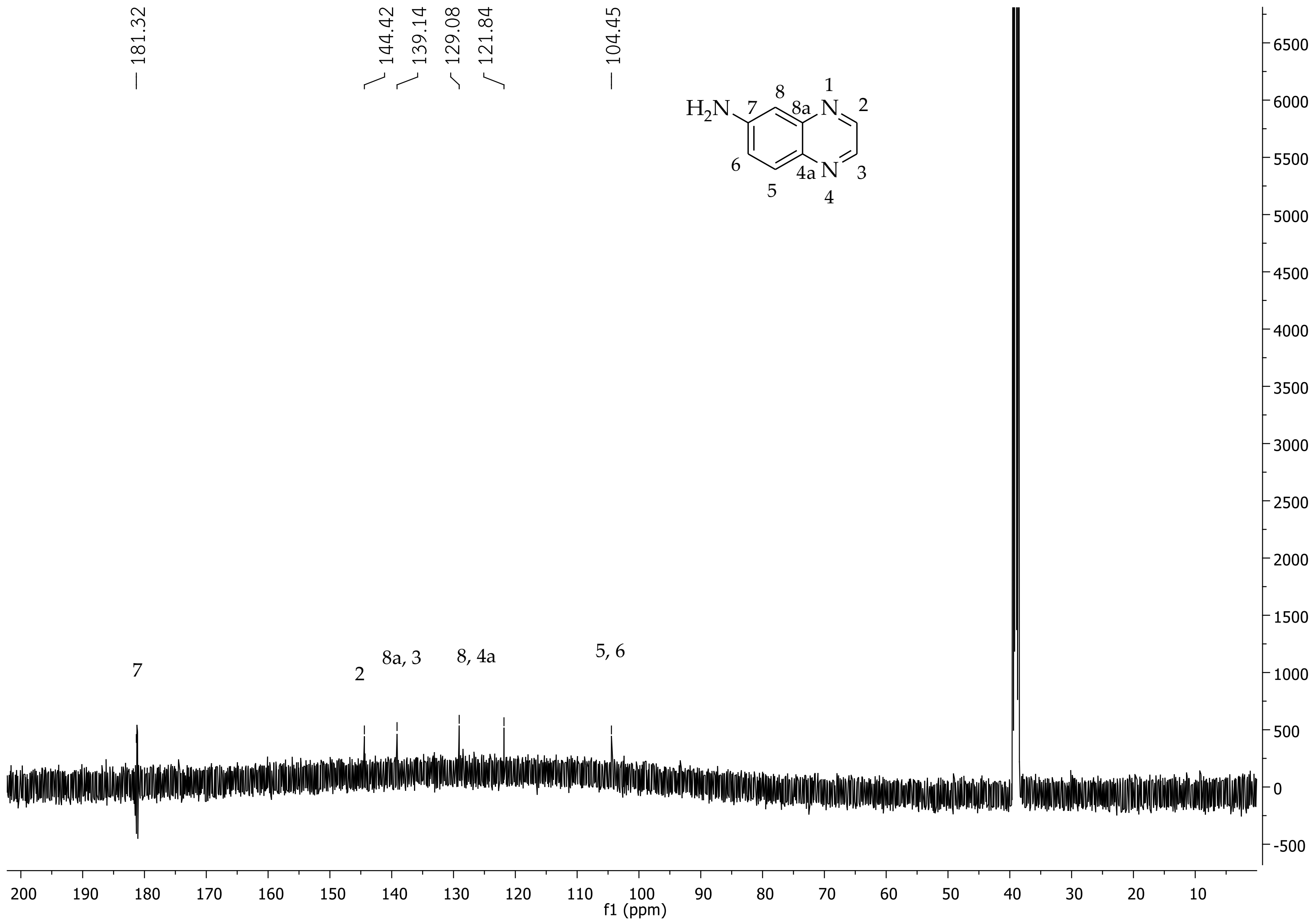

Figure 20. ^{13}C-NMR spectra of Quinoxalin-6-amine (**6a**).

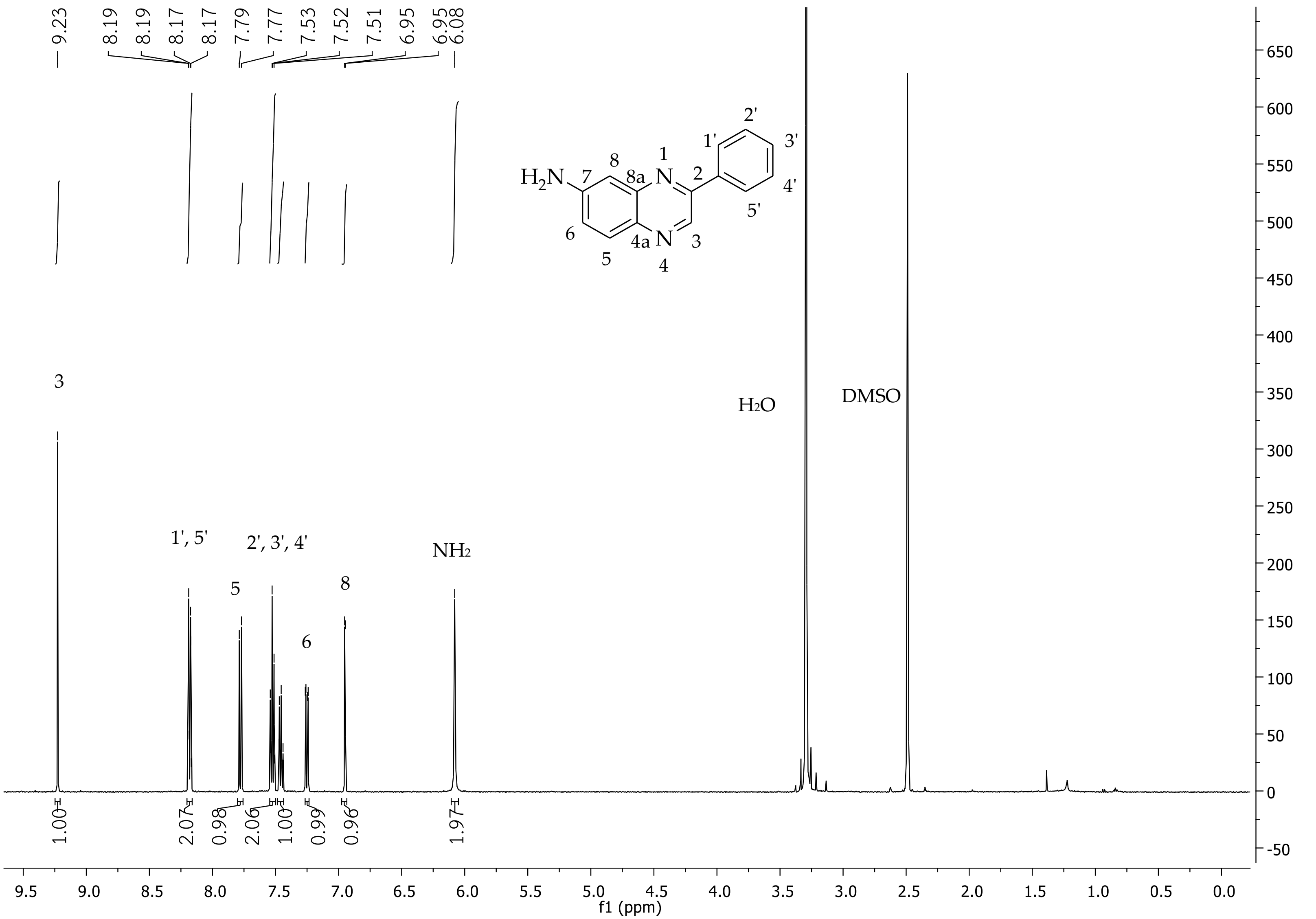

Figure 21. ^{1}H-NMR spectra of 3-Phenylquinoxalin-6-amine (**6b**).

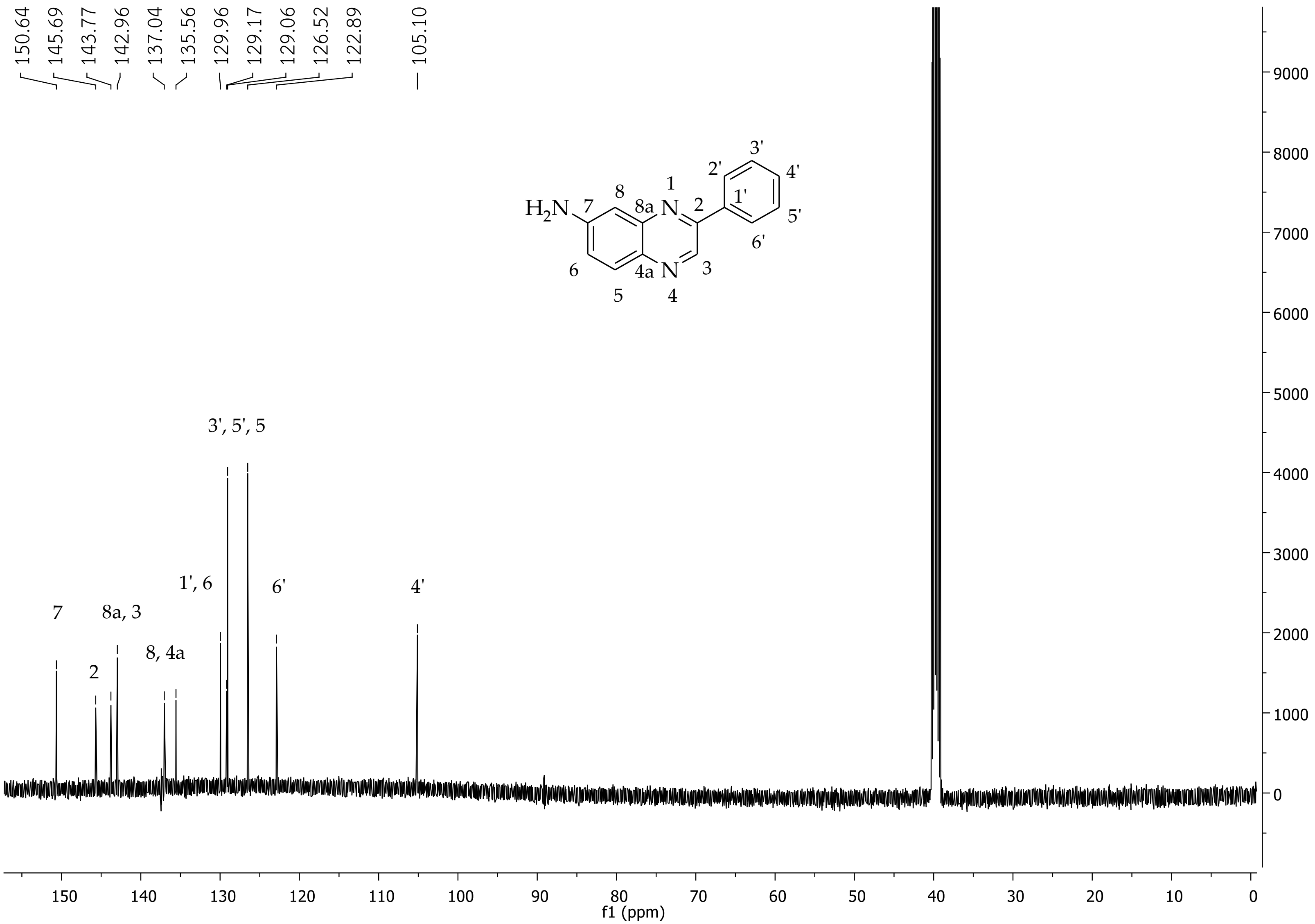

Figure 22. ^{13}C-NMR spectra of 3-Phenylquinoxalin-6-amine (**6b**).

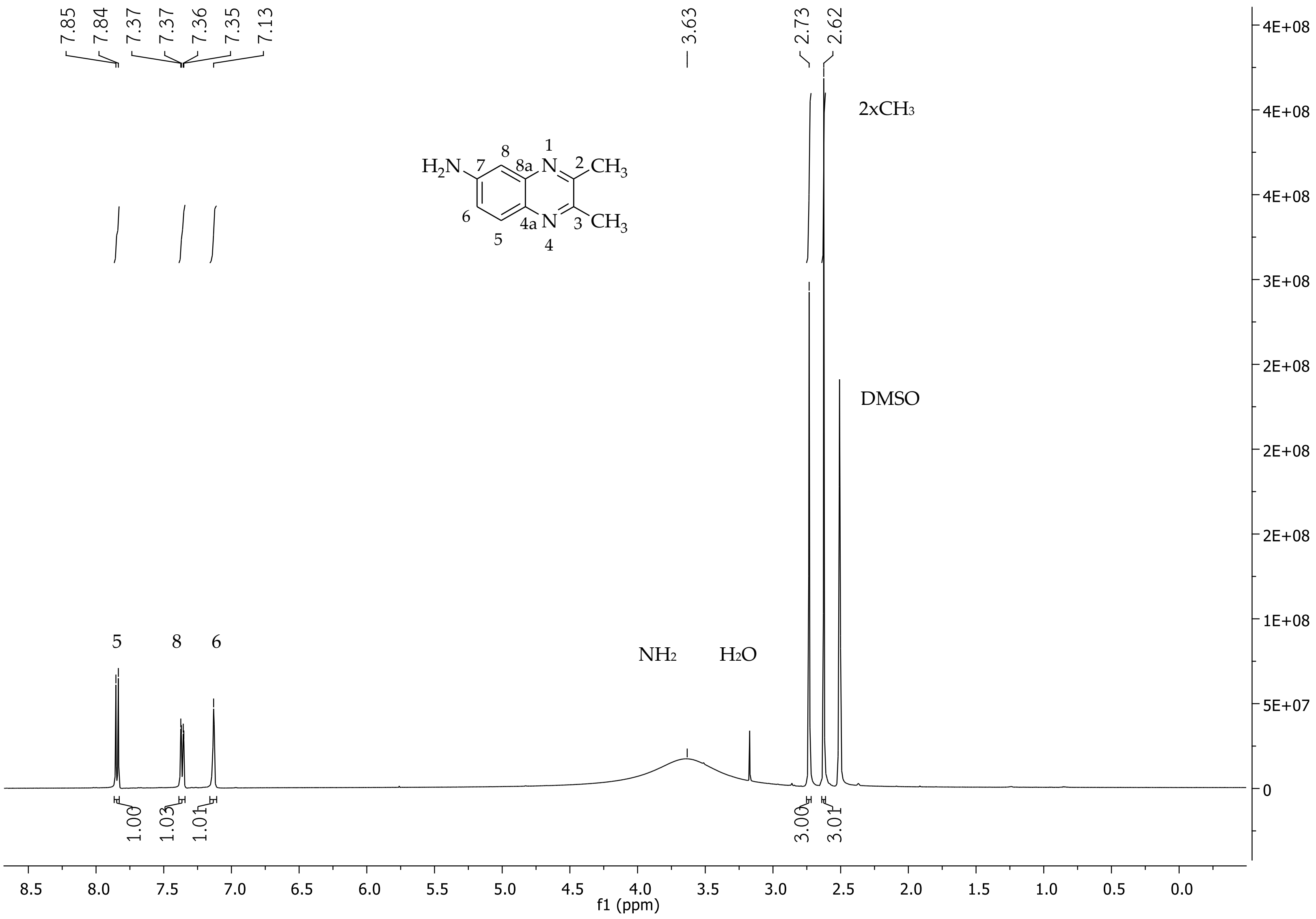

Figure 23. ^{1}H-NMR spectra of 2,3-Dimethylquinoxalin-6-amine (**6c**).

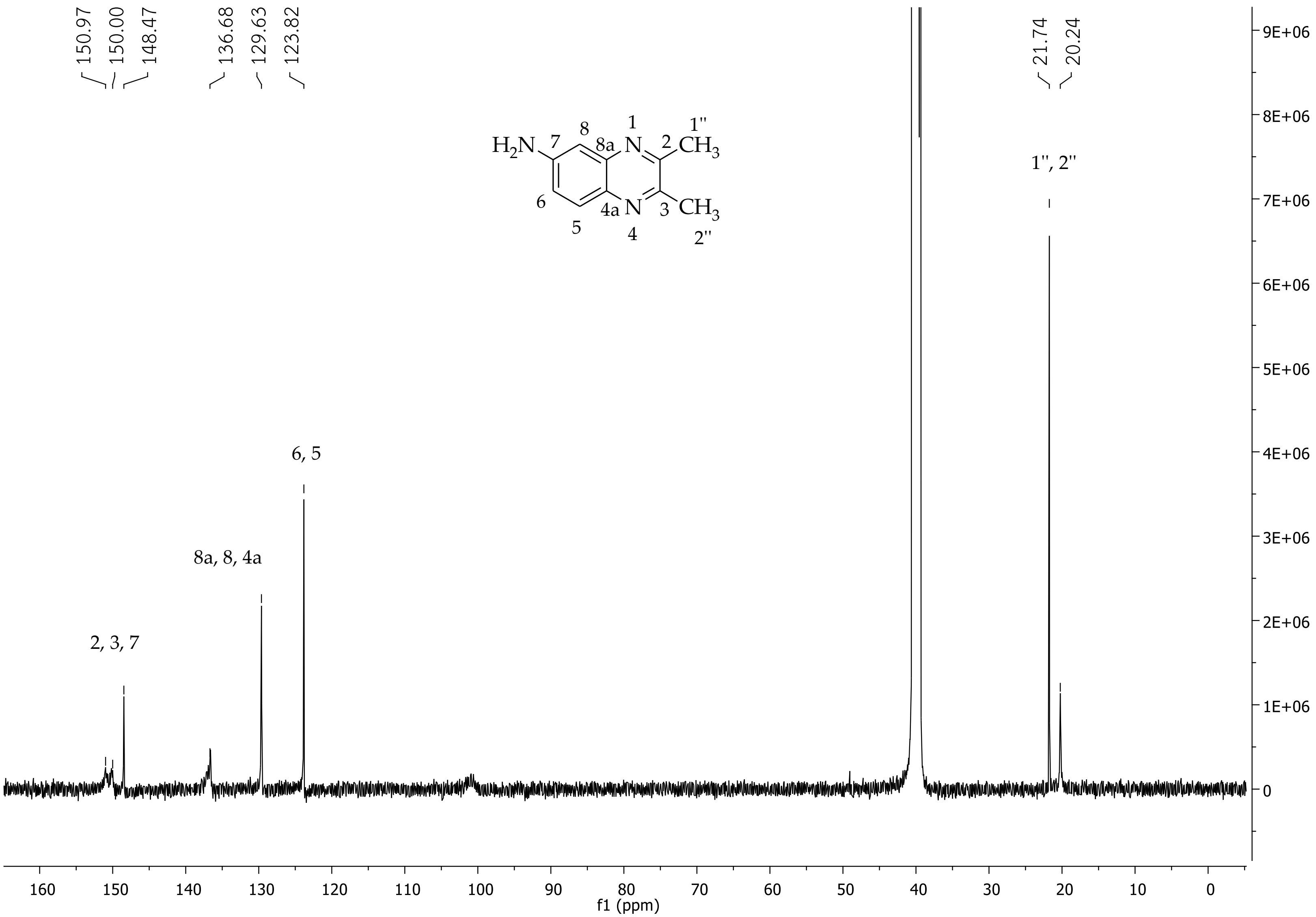

Figure 24. ^{13}C-NMR spectra of 2,3-Dimethylquinoxalin-6-amine (**6c**).

Parameter: Nonlinear regression, Equation: One site competition

Y=Bottom + (Top-Bottom)/(1+10^(X-LogEC50)); Competition of radioligand binding to single binding site identified with a radiolabelled ligand.; Enter data with X= log(Concentration) and Y=binding.

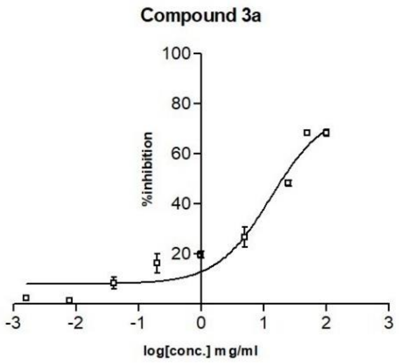

$R^2 = 0.9543$

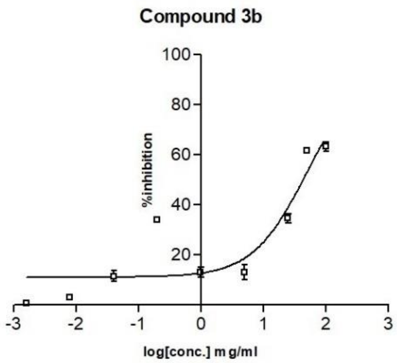

$R^2 = 0.9135$

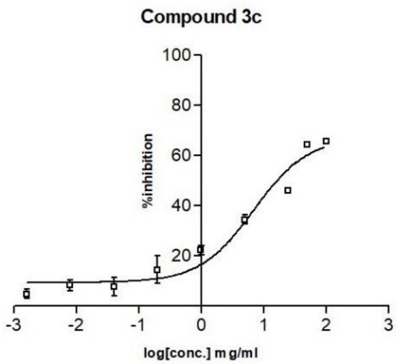

$R^2 = 0.9551$

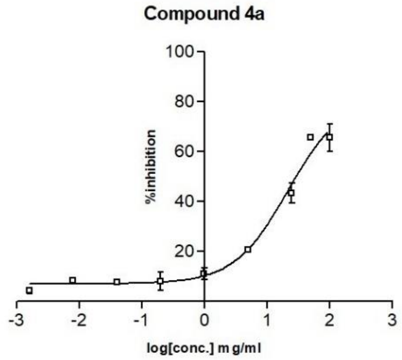

$R^2 = 0.9772$

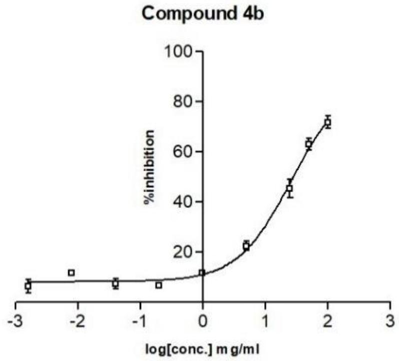

$R^2 = 0.9889$

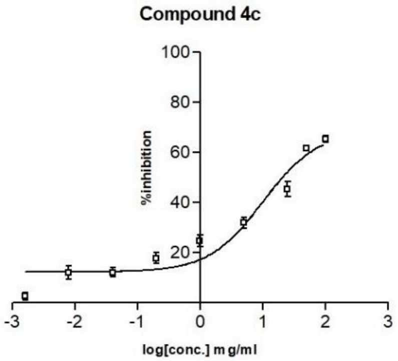

$R^2 = 0.9382$

Figure 25. IC_{50} curves for the inhibition of *Hu*AChE by quinoxaline derivatives (**3a-4c**)

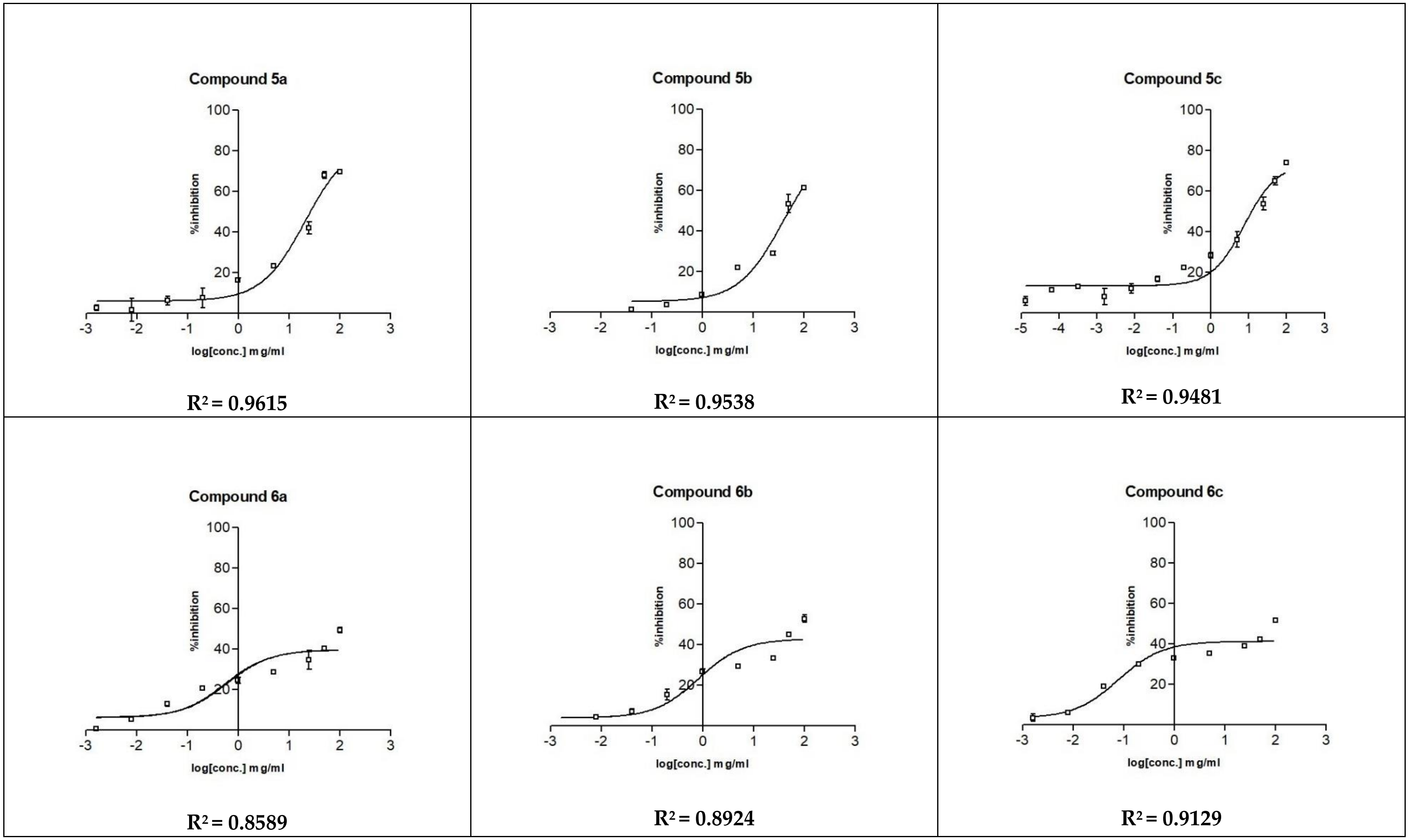

Figure 26. IC_{50} curves for the inhibition of *Hu*AChE by quinoxaline derivatives (**5a-6c**)

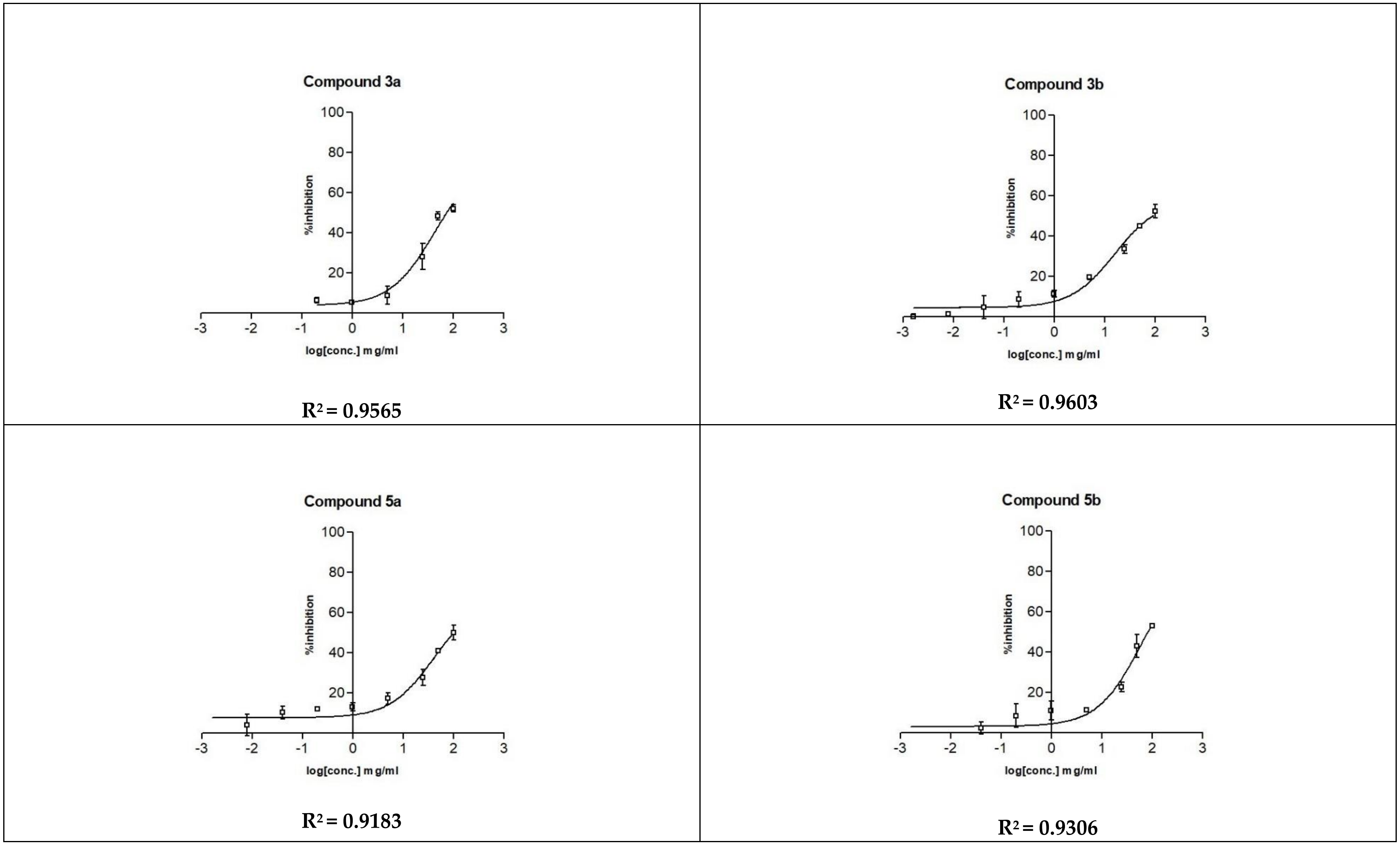

Figure 27. IC_{50} curves for the inhibition of *eq*BChE by quinoxaline derivatives (**3a-3b, 5a-5b**).

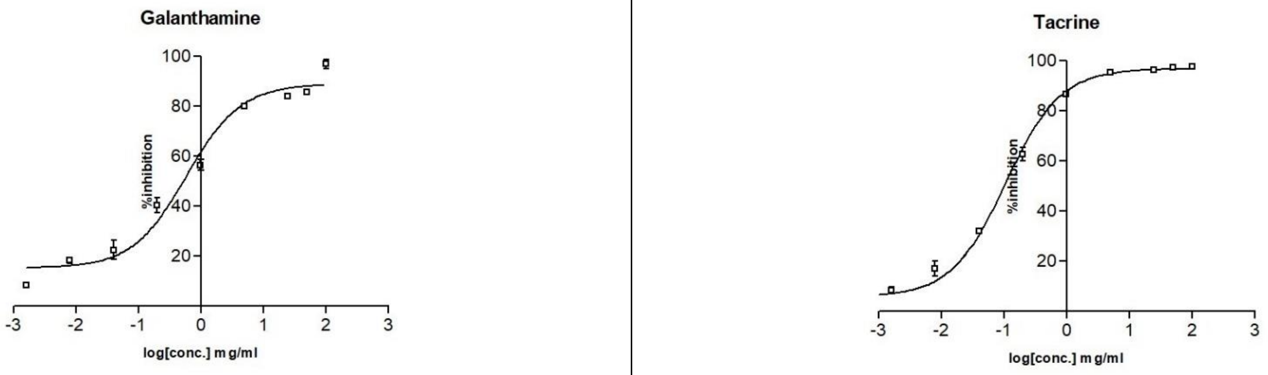

$R^2 = 0.9729$ $R^2 = 0.9955$

Figure 28. IC_{50} curves for the inhibition of *Hu*AChE by galanthamine and tacrine

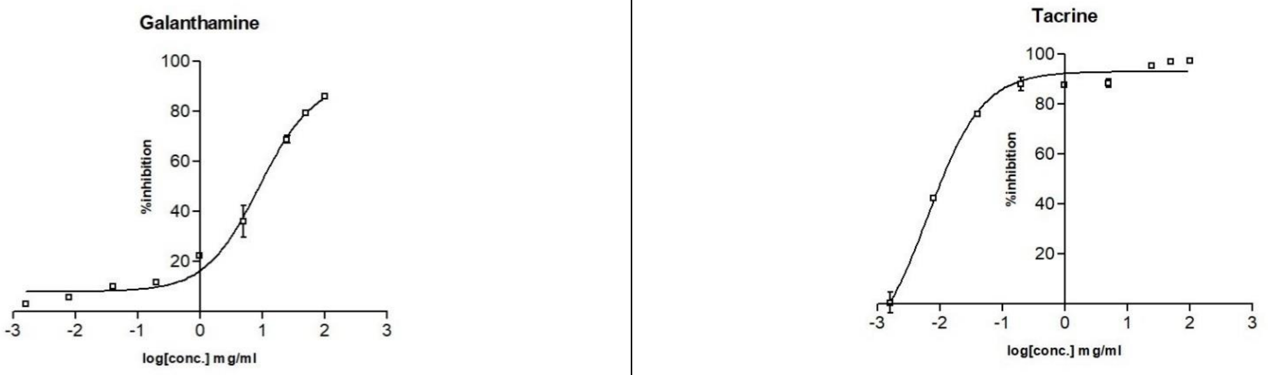

$R^2 = 0.9887$ $R^2 = 0.9872$

Figure 29. IC_{50} curves for the inhibition of *eq*BChE by galanthamine and tacrine.

In PAS of active site of *Hu*AChE enzyme: 2D schematic diagram of docking model of compounds **6a**, **6b**, **6c** and thioflavinT with *Hu*AChE.

<table>
<tr><th>Compound 6a</th><th>Compound 6b</th><th>Compound 6c</th></tr>
<tr>
<td>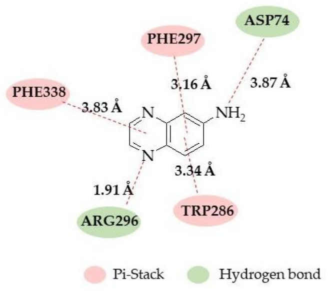
</td>
<td>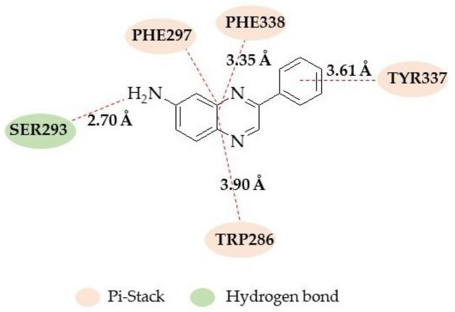
</td>
<td>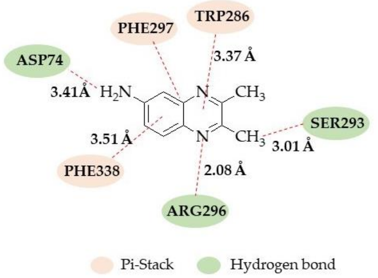
</td>
</tr>
<tr>
<td>Compound 6a-HuAChE complex showed affinity of binding energy of -5.68 kcal/mol. The quinoxaline ring can interact with Trp286, Phe297 and Phe338 with π-π interaction and exhibited hydrogen bonding with Asp74 and Arg296.</td>
<td>Compound 6b-HuAChE complex, showed affinity of binding energy of -7.35 kcal/mol. Pyrazine ring of quinoxaline was observed to bind to PAS via a π-π interaction with Trp286, Phe297 and Phe338. Amino group interacted with Ser293 showed hydrogen bond and benzyl group displayed hydrophobic interaction with Tyr337.</td>
<td>compound 6c-HuAChE complex showed affinity of binding energy of -6.32 kcal/mol. Quinoxaline ring showed π-π interaction with Trp286, Phe297 and Phe338. Nitrogen atom of the pyrazine ring showed hydrogen bond with Arg296. Amino group interacted with Asp74 via hydrogen bond. Methyl group displayed hydrogen bond with Ser293.</td>
</tr>
<tr><th colspan="2">ThioflavinT</th><td></td></tr>
<tr>
<td colspan="2">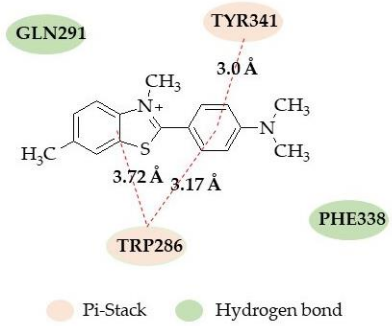
</td><td></td>
</tr>
<tr>
<td colspan="2">Thioflavin T-HuAChE complex, showed affinity of binding energy of -8.29 kcal/mol. Benzothiazol ring was observed to bind to PAS via a π-π interaction with Trp286. Benzyl group displayed π-π interaction with Trp286 and Tyr341.</td><td></td>
</tr>
</table>

In the area of PAS of *Hu*AChE, **(a)** 3D docking model of compound **27** complex with *Hu*AChE. **(b)** 3D docking of compound **21** complex with *Hu*AChE.

**(a): Compound 27**	**(b): Compound 21**
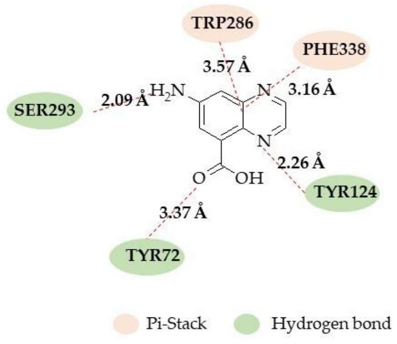	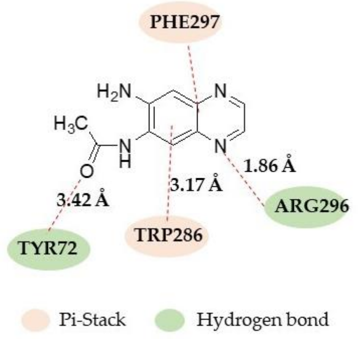
Binding Energy: -4.80 kcal/mol	Binding Energy: -7.13 kcal/mol
Compound **27**-*Hu*AChE complex (**a**), The quinoxaline ring showed π-π stack interaction with Trp286 and Phe338. Nitrogen atom of pyrazine ring formed hydrogen bond with Tyr124. Amino group on quinoxaline ring exhibited hydrogen bond with Ser293 and carboxylic group displayed hydrogen bond with Tyr72.	Compound **21**-*Hu*AChE complex (**b**), the quinoxaline ring showed π-π stack interaction with Trp286 and Phe297. The nitrogen atom of the pyrazine ring showed hydrogen bond with Arg296. Carbonyl group on quinoxaline ring exhibited hydrogen bond with Tyr72.
